# Brown Seaweeds and Their Bioactive Compounds in Type 2 Diabetes: Mechanisms Underlying Metabolic Regulation

**DOI:** 10.3390/ijms27114753

**Published:** 2026-05-25

**Authors:** Un Ju Jung, Sang Ryong Kim

**Affiliations:** 1 Department of Food Science and Nutrition, Pukyong National University, 45 Yongso-ro, Nam-gu, Busan 48513, Republic of Korea; 2 BK21 FOUR KNU Creative BioResearch Group, School of Life Science and Biotechnology, Kyungpook National University, 80 Daehak-ro, Buk-gu, Daegu 41566, Republic of Korea

**Keywords:** type 2 diabetes, brown seaweeds, bioactive compounds, glycemic control, multi-organ metabolic pathways

## Abstract

Type 2 diabetes (T2D) is a multifactorial metabolic disorder characterized by chronic hyperglycemia, insulin resistance, and progressive β-cell dysfunction. Chronic hyperglycemia in T2D causes multi-organ and systemic damage, leading to a wide range of complications, including cardiovascular disease and metabolic dysfunction-associated steatotic liver disease (MASLD). Brown seaweeds are increasingly recognized as promising marine-derived functional foods because they contain structurally unique bioactive compounds, including fucoidan, alginate, phlorotannins, and fucoxanthin. A growing body of evidence suggests that these compounds influence glucose homeostasis through multiple mechanisms, including improvement of pancreatic β-cell function, regulation of gut-mediated metabolic processes, and modulation of glucose metabolism and insulin signaling in the liver, adipose tissue, and skeletal muscle, and attenuation of chronic inflammation and oxidative stress. Brown seaweed-derived bioactive compounds have also been reported to improve abnormal lipid metabolism, a key pathological process implicated in metabolic disorders associated with T2D, including MASLD. This review provides an overview of the antidiabetic potential of brown seaweeds, with a particular focus on the mechanisms of action of their major bioactive compounds, including fucoidan, alginate, phlorotannins, and fucoxanthin.

## 1. Introduction

Type 2 diabetes (T2D) is a complex and progressive metabolic disease characterized by chronic hyperglycemia resulting from insulin resistance, impaired insulin secretion, and dysregulated glucose homeostasis across multiple organs, including the liver, skeletal muscle, adipose tissue, and intestine [1]. The global prevalence of T2D continues to rise at an alarming rate, driven by population aging, sedentary lifestyles, and increasing obesity [2]. According to the World Health Organization, an estimated 830 million people worldwide are living with diabetes, and its prevalence has risen steadily over the past three decades, with T2D accounting for the vast majority of cases [3]. As a result, T2D has emerged as a major global health challenge, affecting hundreds of millions of individuals and imposing substantial clinical and economic burdens worldwide.

Traditionally, T2D management has focused primarily on achieving glycemic control to prevent hyperglycemia-driven microvascular complications, such as retinopathy and neuropathy, alongside the management of macrovascular risk, including cardiovascular disease (CVD) [4]. However, current therapeutic strategies increasingly adopt a multifactorial approach that integrates glycemic control with the management of body weight, cardiovascular risk factors, comorbidities, and diabetes-related complications [4]. In particular, a recent ADA consensus report has drawn attention to metabolic dysfunction-associated steatotic liver disease (MASLD) as a common but under-recognized hepatic comorbidity in people with prediabetes or T2D, especially those with coexisting obesity [5]. T2D and MASLD are closely linked through several common pathophysiological mechanisms, including insulin resistance, chronic low-grade inflammation, oxidative stress, ectopic lipid accumulation, and adipose tissue dysfunction, which also contribute to broader cardiometabolic dysfunction [6,7]. Therefore, there is growing interest in multi-target strategies that improve glycemic control and ameliorate T2D-associated MASLD and broader cardiometabolic dysfunction.

Alongside pharmacological advances, dietary and nutritional strategies have attracted increasing attention as complementary approaches for the prevention and management of T2D [8]. Among these, marine-derived functional foods, particularly brown seaweeds, have emerged as potential modulators of metabolic health [9]. Brown seaweeds, such as *Undaria pinnatifida* (Wakame), *Saccharina japonica* (Kombu), *Sargassum* spp., and *Fucus vesiculosus* (Bladderwrack), are rich sources of structurally unique bioactive compounds, including sulfated polysaccharides (e.g., fucoidan and alginate), phlorotannins, and fucoxanthin, that are absent or extremely rare in terrestrial plants [10,11]. Epidemiological observations and experimental studies suggest that habitual seaweed consumption is associated with improved metabolic profiles, including lower postprandial glycemia, enhanced insulin sensitivity, and reduced cardiometabolic risk [12,13,14,15]. These beneficial effects have been attributed, at least in part, to seaweed-specific components, such as fucoidan, alginate, phlorotannins, and fucoxanthin, which exhibit diverse biological activities relevant to glucose and lipid metabolism, insulin signaling, inflammation, oxidative stress, and gut-mediated metabolic regulation [12,16,17].

Previous reviews have summarized the antidiabetic potential of marine brown algae or assessed the effects of brown seaweed interventions on glycemic outcomes in humans [18,19]. However, these reviews have focused mainly on general antidiabetic activities or clinical glycemic outcomes, whereas an integrated mechanistic overview of major brown seaweed-derived bioactive compounds across multiple metabolic organs, together with their bioavailability, safety, and clinical relevance in T2D, remains limited.

In this comprehensive narrative review, we provide an overview of the antidiabetic effects of brown seaweeds and their bioactive compounds, with a particular focus on the underlying mechanisms of four major bioactive components (fucoidan, alginate, phlorotannins, and fucoxanthin) in T2D. We summarize current evidence on their effects on pancreatic β-cell function and insulin secretion, gut-mediated metabolic regulation, and glucose and lipid metabolism, and insulin signaling in the liver, adipose tissue, and skeletal muscle. In addition, we discuss available evidence on their bioavailability, safety, and clinical relevance.

## 2. Brown Seaweeds and Their Bioactive Compounds

Brown seaweeds (Phaeophyceae) are multicellular marine macroalgae characterized by the presence of the xanthophyll pigment fucoxanthin that contributes to their distinctive brown-olive coloration and distinguishes them from red (Rhodophyta) and green (Chlorophyta) algae [20]. They are widely distributed in temperate and cold coastal waters and have traditionally been consumed in East Asian diets, especially in Korea, Japan, and China [21]. Among them, *U. pinnatifida*, *Saccharina japonica* and *Ecklonia cava* are among the most studied because of their high biomass, extensive cultivation, and long history of use as foods and traditional remedies [21,22]. In addition, species within the genus *Sargassum*, as well as *F. vesiculosus*, and *Ascophyllum nodosum*, have been intensively investigated as rich sources of bioactive compounds with potential applications in functional foods and nutraceuticals [22,23,24].

Brown seaweeds are not only rich in macronutrients (e.g., carbohydrates, proteins, and minerals) but also serve as a reservoir of structurally unique bioactive compounds that are largely absent or rare in terrestrial plants [22]. This review primarily emphasizes well-characterized and extensively studied components that have demonstrated antioxidant, anti-inflammatory, antidiabetic, and cardiometabolic benefits, including polysaccharides (e.g., alginate and fucoidan), phlorotannins (largely unique to brown algae), and fucoxanthin. Although not discussed in detail in this review, other minor bioactive components, such as peptides and lipid-derived metabolites, have also been suggested to contribute to the metabolic effects of brown seaweeds [22].

### 2.1. Major Brown Seaweed Species

This section focuses on widely consumed edible brown seaweed species, as well as functionally important species that have been extensively investigated for their bioactive composition and metabolic health-related benefits.

*U. pinnatifida* is one of the most widely consumed edible brown seaweeds and is a staple ingredient in Korean and Japanese diets. It is rich in fucoxanthin and contains major brown-algal polysaccharides, including fucoidan and alginate [22,25]. It has traditionally been regarded as a health-promoting food with physiological benefits related to circulation, digestion, skin health, and reproductive function. Experimental and human studies have also demonstrated beneficial effects on postprandial glucose regulation, insulin sensitivity, and lipid metabolism, supporting growing interest in wakame as a functional brown seaweed in T2D [26,27].

*Saccharina japonica* is another extensively consumed and commercially cultivated species [28]. It has been used for culinary and medicinal purposes in China, Japan, and Korea for more than a thousand years. It is characterized by high iodine content and substantial levels of polysaccharides, particularly fucoidan, alginate, and laminarin [21,29]. *S. japonica* has attracted considerable attention as a functional edible seaweed with multiple physiological activities. A growing body of experimental studies suggests beneficial roles in antioxidant defense, inflammation modulation, immune function, and metabolic health [29]. Its viscous polysaccharide matrix has been implicated in delayed gastric emptying and reduced intestinal glucose absorption, and its fucoxanthin and fucosterol content has been linked to improvements in lipid metabolism and hepatic steatosis in preclinical models [29].

*E. cava* is an edible marine brown seaweed distributed along the coastal waters of Korea and Japan [22]. It is widely recognized as a rich source of phlorotannins rather than a staple food, and is more commonly utilized in functional foods and nutraceutical applications [30]. This species is particularly rich in polyphenolic phlorotannins, including dieckol, 6,6′-bieckol, and phlorofucofuroeckol, and also provides polysaccharides (e.g., fucoidan and alginate), various minerals, and sterol derivatives (e.g., fucosterol, ergosterol, and cholesterol) [31,32,33]. These compounds have been investigated for their antidiabetic, anti-inflammatory, and antioxidant properties [34,35,36,37,38].

The genus *Sargassum* comprises over 300 species of brown seaweeds and has attracted increasing attention for its potential metabolic effects [39]. Although certain *Sargassum* species, such as hijiki (*S. fusiforme*), have been traditionally consumed in Japan, their use as daily foods remains limited due to safety concerns associated with high levels of inorganic arsenic [40]. However, *Sargassum* species are nutritionally rich and contain key bioactive components, including polyphenols and sulfated polysaccharides [39]. Accumulating studies have demonstrated that these compounds exhibit various biological activities, including antioxidant, anti-inflammatory, antimicrobial, antiviral, neuroprotective, anticoagulant, immunomodulatory, and hepatoprotective effects [39]. Moreover, recent studies have increasingly highlighted their roles in modulating glucose and lipid metabolism, insulin resistance, and obesity-related pathways, supporting their potential as valuable resources for pharmaceutical and nutraceutical applications [41].

*F. vesiculosus* and related Fucus species have a long history of use in traditional European herbal medicine. These species are particularly rich in phlorotannins and have been extensively investigated for their inhibitory effects on carbohydrate-digesting enzymes (e.g., α-amylase and α-glucosidase), as well as their antioxidant and anti-inflammatory activities [24,42]. Although *Fucus* species are less frequently consumed as staple foods than wakame or kombu, accumulating evidence suggests that their polyphenol-rich constituents may exert beneficial effects on T2D-related metabolic dysfunction.

*A. nodosum* is a brown seaweed distributed along the North Atlantic coasts of Europe, northern Canada, and the United States [43]. It is rarely consumed as a staple food but has been widely used as a dietary supplement. This species is rich in bioactive components, including sulfated polysaccharides, phlorotannins, and a wide range of minerals. A growing body of evidence indicates that *A. nodosum* exhibits diverse biological activities, including antioxidant, anti-inflammatory, DNA-protective, neuroprotective, and anticancer effects [43]. Notably, extracts of *A. nodosum* in combination with *F. vesiculosus* have been shown to modulate postprandial insulin responses and improve insulin sensitivity in humans [44]. In addition, clinical studies using nutraceutical formulations containing these seaweeds in combination with other components (e.g., chromium picolinate) have reported improved glycemic control in patients with T2D, although the effects cannot be attributed solely to the seaweed components due to the combined formulation [45].

### 2.2. Key Bioactive Compounds in Brown Seaweeds

**Figure 1:**
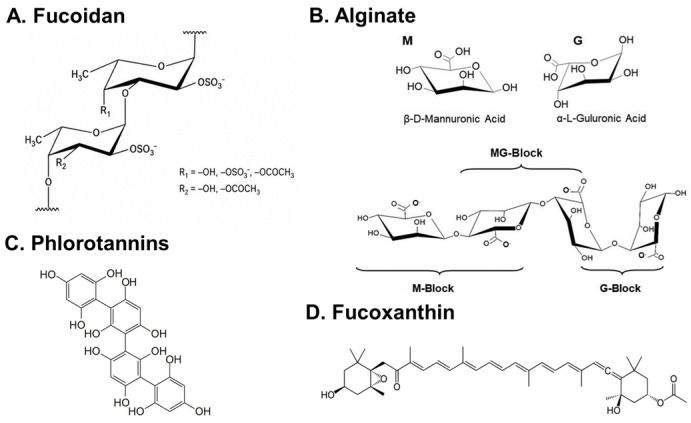
Representative chemical structures of major bioactive compounds derived from brown seaweeds. (**A**) Fucoidan, a sulfated fucose-rich polysaccharide (**B**) Alginate, a linear polysaccharide composed of β-D-mannuronic acid (M) and α-L-guluronic acid (G) residues, arranged as homopolymeric M- and G-blocks and heteropolymeric MG-blocks (**C**) Phlorotannins, polyphenolic compounds derived from phloroglucinol units (**D**) Fucoxanthin, a brown seaweed carotenoid characterized by an allenic bond, epoxide group, hydroxyl groups, and a conjugated carbonyl system.

The metabolic effects of brown seaweeds are largely associated with a diverse array of structurally unique bioactive compounds that are rare or absent in terrestrial plants. These compounds can be broadly categorized into carotenoids, polyphenols, polysaccharides, phytosterols, and other lipid-derived compounds, all of which contribute distinct but often complementary biological activities [21]. This section primarily focuses on key bioactive compounds derived from brown seaweeds, including fucoidan, alginate, phlorotannins, and fucoxanthin (Figure 1), which have been reported to exhibit potential antidiabetic effects [21,46,47,48,49,50,51,52,53,54,55,56,57,58,59,60,61,62,63,64].

#### 2.2.1. Polysaccharides

Polysaccharides are among the most abundant and well-studied bioactive constituents of brown seaweeds and are widely recognized as key contributors to their metabolic functions and health benefits [21]. They account for approximately 4–76% of the total dry mass of brown seaweeds [21]. Major polysaccharides, including alginates, fucoidans, and laminarin, are commonly found across various brown seaweeds such as *U. pinnatifida*, *Saccharina japonica*, *E. cava*, *Sargassum* spp., *F. vesiculosus*, and *A. nodosum*.

Alginate is one of the major polysaccharides abundantly present in brown seaweeds and accounts for a significant proportion of the cell wall matrix [47]. Alginate is a linear anionic polysaccharide composed of (1→4)-linked β-D-mannuronic acid (M) and α-L-guluronic acid (G). These residues are organized into homopolymeric M-blocks, G-blocks, and heteropolymeric MG-blocks, resulting in diverse structures, molecular weight (Mw)s, and physicochemical properties [47]. Due to its high viscosity and gel-forming properties, alginate can slow gastric emptying and intestinal nutrient absorption, thereby attenuating postprandial glucose responses [46,48]. Although its direct metabolic signaling mechanisms are not yet well understood, alginate may exert metabolic effects by modulating the gut microbiota. Alginate oligosaccharides can be fermented by the gut microbiota, leading to increased production of short-chain fatty acid (SCFA), which are known to contribute to improved insulin sensitivity and metabolic homeostasis [49,65].

Fucoidan is a complex, water-soluble polysaccharide primarily found in brown seaweeds [66]. It is composed mainly of α-L-fucose residues with sulfate groups, and its structural characteristics, including the degree of sulfation, monosaccharide composition, and branching patterns, vary among species and extraction conditions [67]. These structural features are closely linked to the diverse biological activities of fucoidan, including anti-inflammatory effects and modulation of the gut microbiota [68,69]. Moreover, accumulating evidence suggests that fucoidan may exert antidiabetic effects through multiple mechanisms, such as modulation of insulin signaling pathways and inhibition of carbohydrate-digesting enzymes, although most evidence is derived from preclinical studies [50,51].

As noted above, although brown seaweeds contain diverse polysaccharides, this review focuses primarily on the effects of alginate and fucoidan on T2D.

#### 2.2.2. Phlorotannins

Phlorotannins are a distinct class of polyphenolic compounds widely distributed in brown seaweeds [52,70]. Unlike tannins found in terrestrial plants, phlorotannins are oligomers and polymers derived from phloroglucinol (1,3,5-trihydroxybenzene) [52,53]. Phlorotannins are commonly classified into six structural subclasses according to the type of linkage between phloroglucinol units and the arrangement of hydroxyl groups [54]. These subclasses include phlorethols, fucols, fucophlorethols, fuhalols, eckols, and carmalols.

Phlorotannins have been reported to have biological activities such as antioxidant, anti-inflammatory, and antimicrobial effects [52,53]. Notably, they also inhibit carbohydrate-digesting enzymes and modulate glucose metabolism, indicating their relevance to T2D [52]. In addition, phlorotannins have been reported to exert a broad range of health benefits, including antiobesity, anticancer, blood pressure-lowering, neuroprotective, and hepatoprotective activities [52]. The biological activities of phlorotannins are closely related to their structural characteristics. In particular, molecular size, degree of polymerization, and the abundance of hydroxyl groups can influence their enzyme-binding and inhibitory properties, as well as their antioxidant activity [54]. Moreover, variations in hydroxyl group position and ether (O-bridge) linkage patterns among phlorotannin units may further affect their biological activity [54].

Brown seaweeds such as *U. pinnatifida*, *Saccharina japonica*, *E. cava*, *Sargassum* species, *F. vesiculosus*, and *A. nodosum* have been reported to contain diverse classes of phlorotannins [52]. The relative abundance and types of phlorotannins vary among species and according to environmental conditions and the harvesting season [55,56].

#### 2.2.3. Fucoxanthin

Fucoxanthin is a major carotenoid found in brown seaweeds and has attracted considerable attention due to its potential metabolic benefits [56]. Fucoxanthin possesses a unique chemical structure characterized by the presence of an allenic bond and an epoxide group [57]. The allenic bond, which represents a rare structural feature in other carotenoids, has been suggested to contribute to potent antioxidant and antiobesity activities of fucoxanthin [57,58,59,60,61]. Moreover, fucoxanthin has been reported to reduce blood glucose and lipid levels, enhance insulin sensitivity, promote fatty acid oxidation, and attenuate hepatic lipid accumulation in various experimental models [62,63,64,71]. However, most of these effects have been demonstrated in animal and in vitro studies, and clinical evidence remains limited. Fucoxanthin is insoluble in water, and its specific molecular structure contributes to its chemical instability, which can limit its bioavailability and effective delivery in vivo [59].

## 3. Mechanisms Underlying the Antidiabetic Effects of Major Bioactive Compounds in Brown Seaweeds

**Figure 2:**
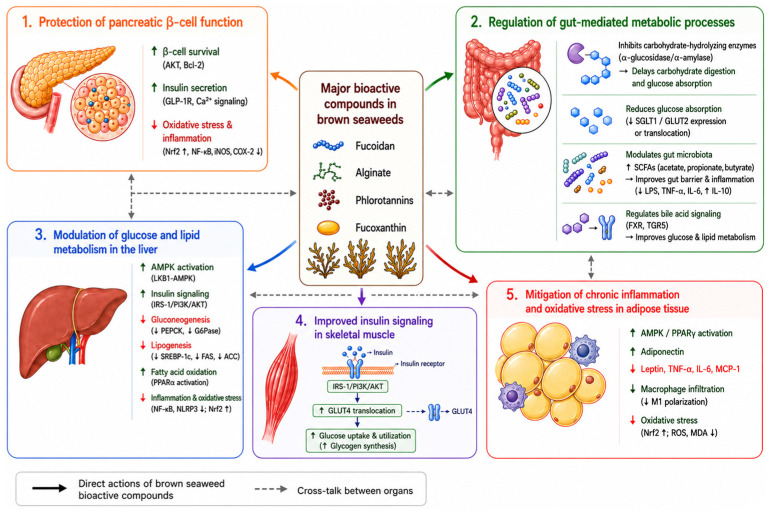
Proposed mechanisms underlying the antidiabetic effects of major bioactive compounds in brown seaweeds. Major bioactive compounds in brown seaweeds, including phlorotannins, fucoidan, alginate, and fucoxanthin, may exert antidiabetic effects through multiple mechanisms, including protection of pancreatic β-cell function, regulation of gut-mediated metabolic processes, modulation of glucose and lipid metabolism, improvement of insulin signaling in target tissues, and mitigation of chronic inflammation and oxidative stress. ACC, acetyl-CoA carboxylase; AKT, protein kinase B; AMPK, adenosine monophosphate-activated protein kinase; Bcl-2, B-cell lymphoma 2; COX-2, cyclooxygenase-2; FAS, fatty acid synthase; FXR, farnesoid X receptor; GLP-1R, glucagon-like peptide-1 receptor; GLUT2, glucose transporter 2; GLUT4, glucose transporter 4; G6Pase, glucose-6-phosphatase; IL-6, interleukin-6; IL-10, interleukin-10; iNOS, inducible nitric oxide synthase; IRS-1, insulin receptor substrate-1; LPS, lipopolysaccharide; MCP-1, monocyte chemoattractant protein-1; MDA, malondialdehyde; NF-κB, nuclear factor kappa B; NLRP3, NLR family pyrin domain containing 3; Nrf2, nuclear factor erythroid 2-related factor 2; PEPCK, phosphoenolpyruvate carboxykinase; PI3K, phosphatidylinositol 3-kinase; PPARα, peroxisome proliferator-activated receptor α; PPARγ, peroxisome proliferator-activated receptor γ; ROS, reactive oxygen species; SCFAs, short-chain fatty acids; SGLT1, sodium-dependent glucose transporter 1; SREBP-1c, sterol regulatory element-binding protein-1c; TGR5, G protein-coupled bile acid receptor 1; TNF-α, tumor necrosis factor-α.

Various bioactive compounds in brown seaweeds exert antidiabetic effects through multiple mechanisms involved in the pathogenesis and complications of T2D. These mechanisms include protection of pancreatic β-cell function, regulation of gut-mediated metabolic processes, modulation of glucose and lipid metabolism and insulin signaling in target tissues, and mitigation of chronic inflammation and oxidative stress (Figure 2). Representative molecular targets and signaling pathways discussed in this review include carbohydrate-digesting enzymes, intestinal glucose transporters, incretin-related signaling, SCFA-related gut metabolic signaling, PTP1B, AMPK, PI3K/AKT, PPARs, GLUT4, and TLR4/NF-κB-related inflammatory pathways. The following subsections summarize the current evidence, highlighting the mechanisms by which brown seaweed compounds may influence glucose and lipid metabolism in T2D.

### 3.1. Modulation of Pancreatic Insulin Secretion and β-Cell Function

Pancreatic β-cells secrete insulin in response to circulating glucose levels, and their dysfunction is a hallmark of T2D [72]. At early stages of the disease, a compensatory increase in β-cell mass and insulin secretion can partially offset rising blood glucose levels [73]. However, prolonged exposure to chronic hyperglycemia promotes inflammatory responses and endoplasmic reticulum (ER) stress, which progressively impair β-cell function and compromise the ability of β-cells to meet insulin demand [74,75]. As the disease advances, sustained β-cell dysfunction and loss of β-cell mass lead to insufficient insulin secretion, ultimately resulting in overt T2D [76,77]. Beyond impaired insulin secretion, β-cell dysfunction in T2D involves defects in glucose sensing, intracellular signaling, insulin synthesis and storage, along with progressive loss of β-cell integrity [78,79,80]. Accordingly, modulation of pancreatic insulin secretion and β-cell function includes not only quantitative changes in insulin output but also coordinated alterations in the molecular, functional, and histological features that govern β-cell responsiveness and survival. Accumulating experimental and clinical evidence suggests that brown seaweed-derived bioactive compounds may modulate pancreatic insulin secretion and preserve or restore β-cell function under diabetic conditions, as discussed below.

#### 3.1.1. Fucoidan

Fucoidan, a sulfated polysaccharide derived from brown seaweeds, has been reported to exert protective effects on pancreatic insulin secretion and β-cell function in diabetes. In streptozotocin (STZ)-treated NIT-1 pancreatic β cells, fucoidan attenuated β-cell apoptosis and restored impaired insulin secretion [81]. These protective effects were mediated through activation of sirtuin-1 (Sirt1), along with upregulation of pancreatic and duodenal homeobox-1 (PDX-1) and glucagon-like peptide-1 receptor (GLP-1R). Consistently, an in vivo study using STZ- and nicotinamide-induced diabetic mice demonstrated that fucoidan administration increased pancreatic insulin content and improved hyperglycemia and glucose tolerance via Sirt1-dependent upregulation of PDX-1 and GLP-1R [81]. PDX-1 is a critical transcription factor for pancreatic development, β-cell differentiation, and maintenance of mature β-cell function and survival, and GLP-1R regulates insulin secretion [82,83,84]. Collectively, these in vitro and in vivo findings indicate that fucoidan modulates pancreatic insulin secretion and preserves β-cell function through coordinated regulation of β-cell survival and insulin-regulatory signaling pathways. Additional experimental evidence supports a role for fucoidan in modulating pancreatic insulin secretion and β-cell function. In Goto-Kakizaki rats, a non-obese T2D model, administration of fucoidan for 13 weeks improved hyperglycemia and restored diabetes-associated reductions in serum insulin levels [84]. In parallel, Jiang et al. [85] demonstrated that fucoidan enhanced glucose-stimulated insulin secretion and increased intracellular cyclic AMP (cAMP) levels in RIN-5F rat insulin-secreting cells. Pharmacological inhibition of cAMP degradation increased fucoidan-induced insulin secretion, whereas suppression of cAMP generation decreased this effect, indicating that fucoidan-stimulated insulin secretion is critically dependent on intracellular cAMP signaling.

In addition to direct effects on insulin secretion and β-cell survival, fucoidan protected pancreatic function and improved glucose metabolism through anti-inflammatory and ER stress-modulating mechanisms [86]. In high-fat diet (HFD)/STZ-induced T2D rats, oral administration of fucoidan for 8 weeks decreased fasting blood glucose and glycated serum protein levels, normalized serum insulin, and increased circulating glucagon-like peptide-1 (GLP-1), while improving insulin sensitivity and reducing postprandial hyperglycemia [86]. These metabolic improvements were accompanied by alleviation of pancreatic damage and enhancement of islet β-cell function. Fucoidan administration also activated the phosphatidylinositol 3-kinase (PI3K)/protein kinase B (PKB, also known as AKT) signaling pathway and suppressed markers of inflammation and ER stress in pancreatic tissue, suggesting that the pancreatic protective effects of fucoidan extend beyond modulation of β-cell secretory processes to include broader regulation of cellular stress and inflammatory signaling under diabetic conditions. Further evidence supporting the protective effects of fucoidan on pancreatic β-cell survival was provided by Wang et al. [87]. Fucoidan derived from *Acaudina molpadioides* inhibited β-cell apoptosis in a high-fat, high-sucrose (HFHS) diet-induced insulin-resistant mouse model through modulation of the mitochondrial apoptotic pathway. In particular, it downregulated mRNA expression of pro-apoptotic genes (Bid, Bax, cytochrome c, caspase-9, and caspase-3), while increasing expression of anti-apoptotic proteins (Bcl-2 and Bcl-xL). These findings indicate that fucoidan inhibits mitochondrial-mediated apoptosis in pancreatic islet cells. Given that β-cell apoptosis is a major contributor to the progressive loss of insulin secretion in T2D, this anti-apoptotic mechanism represents a critical pathway by which fucoidan can preserve β-cell function.

Supporting these experimental findings, a clinical study involving overweight or obese adults reported that fucoidan administration (500 mg/day for 3 months) modulated insulin secretion and improved insulin resistance, suggesting that the insulinotropic effects of fucoidan observed in experimental models may extend to humans [88]. These findings collectively suggest that fucoidan exerts multifaceted protective effects on pancreatic β-cells by integrating anti-apoptotic and insulinotropic mechanisms.

#### 3.1.2. Alginate

Alginate has been widely explored in diabetes research primarily as a biomaterial that shapes the β-cell microenvironment rather than as a metabolizable nutrient that directly triggers canonical β-cell stimulus-secretion signaling. To date, evidence that alginate itself directly modulates intracellular β-cell signaling pathways remains limited. Instead, most evidence supports its indirect modulation of insulin secretory capacity via extracellular and physicochemical cues. Consistent with this view, alginate-based encapsulation has been shown to preserve islet viability and long-term glucose responsiveness of C-peptide secretion in vitro [89].

In contrast, specific alginate chemistries can impair β-cell secretory function. Alginate–catechol cross-linking markedly reduced glucose-stimulated insulin secretion in murine islets without overt cytotoxicity, indicating that material–cell interactions and/or mass transport properties may perturb insulin secretory function [90]. Notably, these contrasting outcomes suggest that the impact of alginate on β-cell function is highly dependent on matrix design. When alginate matrices are designed to resemble the extracellular matrix, β-cell spheroids embedded in softer, arginine–glycine–aspartic acid-conjugated hydrogels exhibit enhanced glucose-dependent insulin secretion [91].

Collectively, current evidence suggests that alginate may influence β-cell function through microenvironmental factors such as diffusion, stiffness, and cell–matrix signaling, rather than through direct intracellular signaling. Although alginate-based systems have been widely investigated in vivo, particularly in islet transplantation models [89,90,91,92], current understanding of how alginate can modulate β-cell function remains largely based on in vitro and ex vivo studies, with limited direct evidence from in vivo models. Further studies are required to elucidate the direct role of alginate itself in modulating pancreatic insulin secretion and β-cell function, independent of matrix-associated effects.

#### 3.1.3. Phlorotannins

Phlorotannins have been investigated for their protective effects on pancreatic β-cells, particularly under glucotoxic conditions. Prolonged hyperglycemia induces chronic oxidative stress, a key driver of glucotoxic injury and functional decline in pancreatic β-cells [93]. In vitro, phloroglucinol, a fundamental structural unit of phlorotannins, protected INS-1 pancreatic β-cells against high-glucose-induced apoptosis by attenuating oxidative stress and mitochondrial dysfunction, thereby preserving β-cell viability under glucotoxic conditions [94]. Similarly, dieckol, another representative phlorotannin, protected insulinoma cells exposed to high glucose by attenuating oxidative stress and subsequent apoptosis [34]. Comparable cytoprotective activity of related phlorotannin derivatives (e.g., 6,6′-bieckol) has also been reported in rat insulinoma cells treated with high glucose, supporting a role for phlorotannins in mitigating glucotoxic β-cell damage [94].

Consistent with these observations, in vivo studies using STZ-induced diabetic rats have shown that phlorotannins extracted from *Cystoseira compressa* attenuated hyperglycemia-associated oxidative stress and reduced pancreatic β-cell injury [95]. Trifuhalol A, a phlorotannin derived from *Agarum cribrosum*, protected pancreatic islets and reduced reactive oxygen species production in alloxan-treated zebrafish larvae [96]. In addition, in an HFD/STZ-induced T2D rat model, a phlorotannin-rich fraction from *Sargassum tenerrimum* attenuated pancreatic histopathological alterations, including β-cell damage and islet structural disruption, concomitant with improved glycemic control [97]. Similarly, in KK-A(y) mice, a widely used model of T2D, polyphenols derived from *Ecklonia kurome* ameliorated hyperglycemia and insulin resistance, and their antidiabetic effects were associated with reduced islet hypertrophy and preservation of pancreatic islet architecture [98], supporting a protective role for phlorotannins in preserving pancreatic β-cell function under T2D conditions.

#### 3.1.4. Fucoxanthin

In a study using T2D *db/db* mice, dietary supplementation with fucoxanthin for 6 weeks prevented abnormal pancreatic histological alterations, ameliorated hyperinsulinemia and insulin resistance, and reduced blood glucose levels [64]. In a separate study employing an HFD/STZ-induced T2D mouse model, a mono-carrier-based encapsulation approach was used to improve the stability and bioavailability of fucoxanthin through nanocomplex formation [99]. Immunofluorescence analysis revealed that mono-carrier encapsulated fucoxanthin nanoparticles restored insulin-positive areas in pancreatic islets, indicating an attenuation of diabetes-induced loss of pancreatic insulin expression. This histological improvement was accompanied by increased protein expression of pancreatic calmodulin-dependent protein kinase (CaMK) and GNAS (encoding the G protein stimulatory α-subunit). In pancreatic β-cells, CaMK and GNAS play key roles as signaling regulators in glucose sensing and insulin secretion [100,101]. Collectively, these molecular and histological changes may contribute to the observed improvements in glucose tolerance and insulin sensitivity following treatment with mono-carrier encapsulated fucoxanthin nanoparticles.

Consistent with these observations, similar protective effects of fucoxanthin on pancreatic function have been reported in another experimental T2D model. In STZ- and nicotinamide-treated mice, fucoxanthin extracted from *Sargassum angustifolium* improved pancreatic islet architecture and increased plasma insulin levels, along with a significant reduction in fasting blood glucose [102]. These effects were comparable to those observed with metformin treatment. Taken together, these findings consistently suggest that fucoxanthin exerts protective effects on pancreatic β-cell structure and function, potentially through the modulation of intracellular signaling pathways involved in glucose sensing and insulin secretion.

Recently, a randomized, double-blind, placebo-controlled clinical trial demonstrated that 12-week supplementation with fucoxanthin (12 mg/day) in patients with metabolic syndrome did not significantly alter fasting blood glucose levels but enhanced insulin secretion [103]. Thus, further well-designed clinical studies are needed to clarify its therapeutic efficacy in patients with T2D.

### 3.2. Gut-Mediated Metabolic Regulation

The small intestine is the main site of carbohydrate digestion and glucose absorption and therefore plays a central role in the regulation of postprandial glycemic responses [104,105]. Complex carbohydrates are initially hydrolyzed by digestive enzymes, primarily α-amylase and α-glucosidase, into absorbable monosaccharides such as glucose and maltose. These monosaccharides are subsequently transported across the intestinal epithelium via sodium-dependent glucose transporter 1 (SGLT1) and facilitative glucose transporter 2 (GLUT2). Attenuation of intestinal carbohydrate digestion and glucose absorption is an important strategy for reducing postprandial hyperglycemia and insulin demand [106].

Accumulating evidence suggests that the expression and activity of SGLT1 and GLUT2 are increased under diabetic or hyperglycemic conditions, contributing to excessive intestinal glucose uptake and impaired glycemic control [107,108]. The trafficking and membrane localization of these transporters are dynamically regulated by intracellular signaling pathways, particularly protein kinase C (PKC). PKC activation enhances glucose uptake through two complementary mechanisms: increasing the apical membrane localization of SGLT1 and promoting the translocation of GLUT2 from the cytoplasm to the brush border membrane [107,109]. The rate of carbohydrate digestion is tightly linked to glucose transporter regulation. Rapid hydrolysis of polysaccharides and disaccharides by α-amylase and α-glucosidase elevates luminal glucose concentrations, which in turn stimulates the upregulation and activation of SGLT1 and GLUT2 to facilitate efficient glucose absorption. This coordinated regulation contributes to the maintenance of glucose homeostasis under physiological conditions. However, in diabetes, accelerated digestion and transporter overexpression synergistically exacerbate hyperglycemia [110]. Therefore, simultaneous inhibition of carbohydrate-digesting enzymes and glucose transporters may exert additive or synergistic effects in mitigating postprandial hyperglycemia.

In addition to carbohydrate digestion and absorption, the gut contributes to glycemic regulation through incretin hormones. During food ingestion, enteroendocrine cells release GLP-1 and glucose-dependent insulinotropic polypeptide (GIP) that synergistically enhance glucose-dependent insulin secretion and improve insulin sensitivity [111]. GLP-1 is an incretin hormone that plays a central role in glucose homeostasis, primarily by stimulating insulin secretion from pancreatic β-cells and suppressing glucagon release from α-cells [112]. It also contributes to glycemic regulation by delaying gastric emptying, increasing satiety, reducing appetite, and protecting β-cell function through activation of GLP-1R-mediated signaling pathways [112,113]. GIP, another incretin hormone, is secreted primarily by K cells located in the duodenum and jejunum, and promotes insulin secretion via binding to the GIP receptor [114]. It has been reported that gut microbiota-derived metabolites modulate incretin secretion and intestinal metabolic regulation [115]. SCFAs, key metabolites produced by gut microbiota (primarily acetate, propionate, and butyrate), have been shown to stimulate GLP-1 secretion in vitro and in vivo, thereby contributing to improved glucose tolerance [65,116]. This effect has been linked, at least in part, to activation of the G protein-coupled receptor FFAR2, providing a receptor-mediated mechanism by which SCFAs can regulate enteroendocrine GLP-1 secretion and contribute to glucose homeostasis [116].

**Figure 3:**
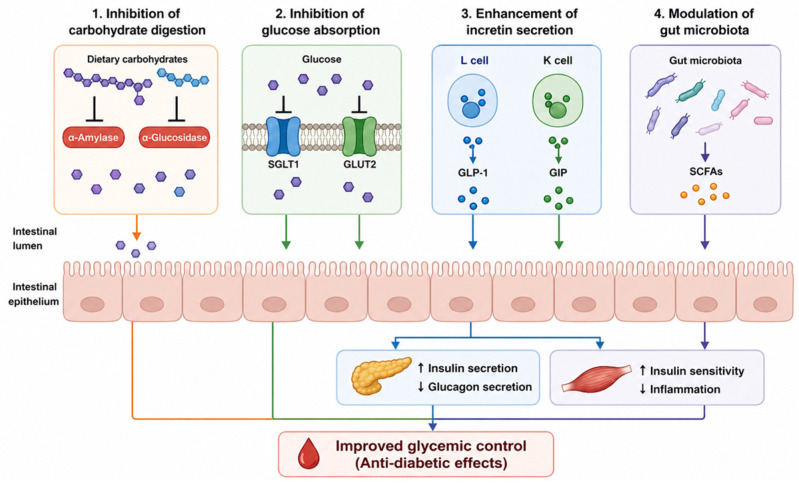
Proposed gut-mediated mechanisms underlying the antidiabetic effects of brown seaweeds and their bioactive compounds. Brown seaweed-derived bioactive compounds may exert antidiabetic effects by inhibiting carbohydrate digestion, reducing intestinal glucose absorption, enhancing incretin secretion, and modulating the gut microbiota and its metabolites.

Collectively, the regulation of intestinal carbohydrate digestion, glucose transporter function, incretin hormone secretion, and gut microbiota-derived signaling forms an integrated gut-mediated regulatory network. Targeting these mechanisms may represent a promising strategy by which brown seaweeds and their bioactive compounds may exert potent antidiabetic effects (Figure 3).

#### 3.2.1. Fucoidan

The gastrointestinal tract is increasingly recognized as a primary site of action for fucoidan-mediated glycemic regulation. Fucoidan, a sulfated polysaccharide from brown seaweeds, possesses diverse structural characteristics such as degree of sulfation and monosaccharide composition that strongly influence its biological activities, particularly its gut-mediated antidiabetic effects.

Previous studies have demonstrated that type II fucoidan, characterized by alternating α-(1→3)- and α-(1→4)-linked fucose units, can inhibit SGLT1-mediated glucose transport in the small intestine, thereby regulating blood glucose levels and alleviating postprandial hyperglycemia [117]. Consistent with this observation, only type II fucoidan, but not type I fucoidan, inhibited glucose transport in Caco-2 monolayers and everted gut sac models and improved both fasting and postprandial hyperglycemia in *db/db* mice [118]. These effects were associated with inhibition of SGLT1 activity and increased circulating GLP-1 levels.

In addition, fucoidan isolated from *Sargassum wightii* directly inhibits carbohydrate-hydrolyzing enzymes, including α-amylase and α-glucosidase [119]. Beyond differences in backbone structure, Mw appears to play a critical role in its inhibitory activity. In general, low-Mw fucoidans show stronger glucose-lowering effects than their high-Mw counterparts [120]. For instance, at equivalent concentrations, a low-Mw sulfated polysaccharide fraction (41.4 kDa) derived from *U. pinnatifida* showed stronger α-glucosidase and α-amylase inhibitory activities than higher-Mw fractions (84.8 and 330.7 kDa) from the same species [121]. Similarly, Kim et al. [68] reported that fucoidan from *A. nodosum* with a lower Mw (637 kDa) inhibited α-amylase, whereas fucoidan from *F. vesiculosus* with a much higher Mw (2351 kDa) showed little or no inhibitory activity despite similar backbone structures. Another in vitro study further supported the α-glucosidase inhibitory activity of low-Mw fucoidan from *Ecklonia maxima* (~10 kDa) [122]. In addition, low-Mw fucoidans or fucoidan-derived oligosaccharides generally exhibited improved solubility and bioavailability, which may contribute to their enhanced biological efficacy [123]. Taken together, low-Mw fucoidans are generally considered to exert stronger hypoglycemic effects.

These Mw-dependent effects of fucoidan on digestive enzyme inhibition were further elucidated by Tang et al. [124], who investigated fucoidan degradation products (FUDPs), comprising low- (<1.5 kDa; LMAF) and moderately low-Mw fractions (1.5–20 kDa; HMAF). Both LMAF and HMAF inhibited α-amylase and α-glucosidase, with HMAF showing greater inhibitory potency. In HFD-fed mice, HMAF more effectively enhanced insulin secretion and improved insulin sensitivity by upregulating GLP-1 and GIP gene expression in the small intestine. Moreover, FUDPs suppressed postprandial glucose elevation by downregulating intestinal SGLT1, GLUT2, and PKCα expression. Collectively, these findings demonstrate that FUDPs (<20 kDa) may regulate postprandial hyperglycemia through coordinated inhibition of carbohydrate-digesting enzymes, attenuation of glucose transporter-mediated absorption, and modulation of incretin signaling, with HMAF exhibiting greater efficacy than LMAF. These results indicate that the bioactivity of fucoidan may depend on an optimal Mw range, in which moderately low-Mw fractions retain sufficient structural features for effective biological interactions, whereas excessively low-Mw fractions may lack the necessary structural complexity for optimal activity.

In addition to directly affecting intestinal glucose digestion and incretin signaling, FUDPs act as soluble dietary fibers that can be fermented by the gut microbiota, leading to SCFA production. Notably, low-Mw fucoidan fractions (<1.5 kDa) preferentially promoted the production of acetic, lactic, and propionic acids, whereas moderately low-Mw fractions (1.5–20 kDa) were more effective in stimulating butyrate production [125]. These SCFAs may further contribute to glycemic regulation by supporting intestinal metabolic signaling pathways, thereby linking microbiota-mediated fermentation to host metabolic control.

Beyond SCFA-mediated effects, fucoidan can exert antidiabetic effects through modulation of gut microbial communities and their metabolic outputs. Zhang et al. [126] demonstrated that fucoidan from *Laminaria japonica* ameliorated hyperglycemia and dyslipidemia in HFD/STZ-induced T2D mice in a dose-dependent manner. Fucoidan supplementation reduced fasting blood glucose and lipid levels while increasing activities of antioxidant enzymes, including catalase and superoxide dismutase, as well as circulating GLP-1 levels. Histological analysis further revealed its protective effects against pancreatic islet necrosis and β-cell damage. Importantly, these metabolic improvements were accompanied by significant remodeling of the gut microbiota, characterized by increased abundances of beneficial genera such as *Lactobacillus* and *Allobaculum*. Metabolomics analyses indicated that fucoidan-induced microbial shifts were associated with altered intestinal metabolite profiles, particularly involving amino acid metabolism, glutathione pathways, and glyoxylate and dicarboxylate metabolism. Collectively, these findings suggest that fucoidan may exhibit prebiotic-like properties and improve glycemic control through microbiota–metabolite interactions.

Consistent with these findings, in HFD/STZ-induced T2D mice, fucoidan from *Sargassum fusiforme* increased gut microbial richness and partially restored microbial community structure toward that of healthy controls [127]. This was accompanied by enrichment of beneficial genera, such as *Bacteroides*, *Faecalibacterium*, and *Blautia*, along with a reduction in potentially harmful bacteria, including *Desulfovibrio* [127]. These microbiota alterations were associated with improvements in glucose metabolism and lipid profiles, further supporting its role in modulating the gut microbiota under diabetic conditions. Zhang et al. [128] also demonstrated that *S. fusiforme* fucoidan improved HFD-induced obesity and insulin resistance in mice by remodeling gut microbial composition and reducing intestinal inflammation. It reduced the abundance of endotoxin-producing bacteria, such as *Desulfovibrionaceae* and *Turicibacter*, while increasing beneficial genera, including *Bacteroides*, *Alistipes*, and *Faecalibacterium*. These shifts were linked to decreased circulating LPS levels, reduced pro-inflammatory cytokines, and enhanced intestinal barrier integrity, thereby contributing to improved insulin resistance. Furthermore, in line with microbiota-mediated regulation of host metabolism, fucoidan alleviated insulin resistance by suppressing colon-derived ceramide biosynthesis, gut microbiota-dependent lipid mediator implicated in metabolic dysfunction [129].

This gut-associated metabolic regulatory potential is further supported by a recent study by Jia et al. [130], in which fucoidan from *Scytosiphon lomentaria* ameliorated colon damage in dietary fiber-deficient mice by preserving epithelial integrity, maintaining goblet cells, and enhancing tight junction protein expression while also suppressing oxidative stress and nuclear factor kappa B (NF-κB)-mediated inflammation. These effects were accompanied by beneficial shifts in the gut microbiota, including increases in *Akkermansia* and *Bacteroides*, as well as altered host lipid metabolism pathways. Although this study used a fiber-deficient rather than a classical diabetic model, it provides mechanistic support for the role of fucoidan in reinforcing the intestinal barrier and attenuating gut-driven inflammation, which may contribute to improved insulin sensitivity and glycemic regulation. Similarly, fucoidan from *U. pinnatifida* alleviated fiber-deficiency-induced intestinal inflammation and lipid dysregulation in mice by modulating mucosal microbiota, increasing SCFA production, and preserving gut barrier integrity [131]. Liu et al. [132] also demonstrated that fucoidan ameliorated hyperglycemia and insulin resistance in HFD/STZ-induced T2D rats by restoring intestinal barrier function. These effects were associated with upregulation of tight junction proteins, including zonula occludens-1 and occludin, along with suppression of intestinal inflammation via inhibition of the toll-like receptor 4 (TLR4)/NF-κB signaling pathway.

Although clinical evidence remains limited, a randomized placebo-controlled trial in patients with T2D demonstrated that 12-week supplementation with a fucoidan-rich beverage (~1.6 g/day) did not significantly improve overall glycemic control [133]. Modest reductions in HbA1c and baseline GLP-1 levels were observed only in a subgroup with normal HOMA-IR (<2.5), suggesting a context-dependent effect. However, these findings should be interpreted with caution due to the crossover design and small sample size.

Taken together, fucoidan and its degradation products may exert multifaceted gut-mediated metabolic effects by modulating intestinal carbohydrate digestion, glucose transport, incretin hormone signaling, gut microbial communities, and their metabolites. Through enhanced crosstalk between the gastrointestinal tract and pancreatic β-cells, fucoidan may contribute to the regulation of glucose absorption and metabolism, thereby potentially improving glucose homeostasis.

#### 3.2.2. Alginate

Alginate is a non-digestible, linear polysaccharide composed of M and G residues that is abundantly found in brown seaweeds [47]. Due to its high viscosity and gel-forming properties in the presence of gastric acid and divalent cations, alginate primarily acts within the gastrointestinal tract rather than through systemic absorption.

Human intervention studies have demonstrated that alginate increases gastric viscosity and delays gastric emptying. A study by Hoad et al. [134] using magnetic resonance imaging showed that alginate-containing beverages prolonged gastric emptying compared with control formulations. This delay in gastric emptying may slow nutrient delivery to the small intestine and attenuate postprandial glycemic responses. In addition, alginate reduced postprandial glucose responses in human trials. Torsdottir et al. [48] reported that sodium alginate supplementation lowered postprandial blood glucose levels in subjects with T2D. Similarly, Harden et al. [135] observed that an ionic-gelling alginate preload attenuated the postprandial glycemic response. These effects may be partly attributed to the fiber-like properties of alginate, an indigestible algal polysaccharide that can function as a thickening agent and may influence intestinal absorption [136].

Alongside these physicochemical effects, alginate has also been reported to exert moderate inhibitory activity against carbohydrate-digesting enzymes. For example, sodium alginate derived from *Ecklonia radiata* and *Sargassum elegans* selectively inhibited α-glucosidase and maltase, with minimal effects on α-amylase and sucrase [137]. In contrast, commercially available sodium alginate exhibited comparatively weak or negligible inhibitory activity. Accordingly, the enzyme inhibitory potential of alginate appears to vary depending on its source and structural characteristics, such as Mw and the M/G ratio.

Regarding gut microbiota interactions, an in vitro study has demonstrated that alginate and its derivatives can be degraded and utilized by human fecal microbiota, along with increased SCFA production [138]. Although direct evidence linking alginate-derived fermentation products to improved glycemic control remains limited, recent in vivo evidence suggests that alginate can modulate gut microbiota composition and microbial metabolite profiles associated with glycemic control [139]. Notably, the antidiabetic effects of alginate derived from *Sargassum fusiforme* were accompanied by significant increases in beneficial bacterial genera (*Lactobacillus*, *Bacteroides*, *Akkermansia*, *Alloprevotella*, *Weissella*, and *Enterorhabdus*), along with decreases in potentially harmful bacteria, such as *Turicibacter* and *Helicobacter*. In parallel, alginate treatment reduced levels of metabolically detrimental microbial metabolites, including branched-chain amino acids and aromatic amino acids, in the colon of HFD/STZ-induced T2D mice.

Collectively, available experimental and limited clinical evidence indicates that alginate may exert antidiabetic effects primarily through gastrointestinal physicochemical mechanisms, including delayed gastric emptying and reduced postprandial glucose absorption. Although microbiota-mediated pathways are biologically plausible, further studies are required to confirm their relevance in T2D.

#### 3.2.3. Phlorotannins

Major phlorotannins, including dieckol, eckol, and phlorofucofuroeckol A, are found primarily in *E. cava* and related species. Among brown seaweed-derived bioactive compounds, phlorotannins have been extensively investigated for their potent inhibitory activity against carbohydrate-digesting enzymes.

One of the most direct and well-established antidiabetic mechanisms of phlorotannins is inhibition of α-glucosidase and α-amylase. Apostolidis et al. [140] demonstrated that phlorotannin-rich extracts from brown seaweeds effectively inhibited both α-glucosidase and α-amylase activities in vitro. Comparable inhibitory effects have been reported for phlorotannin-rich fractions from Alaskan seaweed [141]. Phlorotannin-rich extracts from *Ecklonia maxima* also exhibited strong α-glucosidase inhibitory activity and notable antiradical properties [142].

The inhibitory effects of individual phlorotannin compounds on carbohydrate-digesting enzymes have also been reported. Phloroglucinol derivatives isolated from *E. cava*, including fucodiphloroethol G, dieckol, 6,6′-bieckol, 7-phloroeckol, and phlorofucofuroeckol A, inhibited rat intestinal α-glucosidase and porcine pancreatic α-amylase in vitro [143]. In separate studies, purified dieckol and phlorofucofuroeckol A from *E. cava* also inhibited α-glucosidase and α-amylase [144,145]. Similar α-glucosidase inhibitory activity was also observed in phlorotannins from *Ecklonia stolonifera* and *Eisenia bicyclis*, including dieckol, 7-phloroeckol, and phlorofucofuroeckol A [146]. Additionally, diphlorethohydroxycarmalol isolated from *Ishige okamurae* exhibited stronger inhibitory activity against both α-glucosidase and α-amylase than acarbose [147]. More recently, trifuhalol A, a phlorotannin derived from *Agarum cribrosum*, also inhibited both α-glucosidase and α-amylase activities [96]. These inhibitory effects are further supported by α-glucosidase inhibition assays and molecular docking analyses [148]. Collectively, these findings indicate that phlorotannins can directly delay carbohydrate digestion and reduce glucose availability for intestinal absorption, an effect that is supported by subsequent in vivo studies.

The in vivo relevance of these enzyme inhibitory effects has also been demonstrated. Dieckol and diphlorethohydroxycarmalol significantly suppressed postprandial blood glucose levels in both STZ-induced diabetic and normal mice following starch administration, suggesting a delay in carbohydrate digestion and absorption [144,147]. Similarly, a commercially available phlorotannin extract derived from *A. nodosum* and *F. vesiculosus* markedly reduced postprandial blood glucose levels and attenuated peak insulin secretion in vivo, further supporting the role of phlorotannins in modulating carbohydrate digestion and glycemic response [149].

Collectively, current in vitro and in vivo evidence suggests that phlorotannins may exert antidiabetic effects primarily through inhibition of carbohydrate-digesting enzymes such as α-glucosidase and α-amylase. However, evidence for their roles in regulating intestinal glucose transporters, incretin hormone secretion, and gut microbiota-derived metabolic pathways remains limited, particularly in T2D models. Further studies are therefore required to elucidate whether phlorotannins can exert broader gut-mediated metabolic effects beyond carbohydrate-digesting enzyme inhibition.

#### 3.2.4. Fucoxanthin

One important mechanism underlying the antidiabetic activity of fucoxanthin involves modulation of gut microbial communities and bile acid-related metabolic signaling. In an HFD-induced obese mice, fucoxanthin extract supplementation (80–320 mg/kg/day for 12 weeks) reduced body weight gain, improved glucose tolerance, and ameliorated dyslipidemia [150]. These metabolic benefits were accompanied by marked alterations in bile acid metabolism and gut microbiota composition. Fucoxanthin increased total bile acid levels and altered the expression of bile acid receptors, including transmembrane G protein-coupled receptor 5 and farnesoid X receptor, suggesting modulation of bile acid receptor-mediated signaling pathways. In addition, 16S rRNA sequencing showed shifts in microbial taxa, including *Lachnospiraceae* and *Oscillospiraceae*, and these changes may contribute to the observed metabolic improvements.

Complementing these in vivo findings, Guo et al. [151] reported the interaction between fucoxanthin and the human gut microbiota using an in vitro simulated digestion–fermentation model. Approximately half of ingested fucoxanthin was not absorbed in the small intestine, indicating that substantial amounts may reach the colon and directly interact with resident microbes. Fucoxanthin supplementation altered gut microbial composition, particularly by increasing the abundance of Bacteroidota and *Parabacteroides*, and enhanced microbial functions associated with glycan metabolism and host endocrine and immune pathways. Metabolomics profiling further revealed significant changes in microbial metabolites, especially bile acids and indole derivatives, supporting the role of fucoxanthin in regulating gut-derived metabolic outputs. Earlier animal studies have also reported microbiota-mediated effects of fucoxanthin, including suppression of obesity- and inflammation-associated taxa and enrichment of butyrate-producing bacteria [152,153]. Although direct evidence for SCFA-mediated effects in diabetic models remains limited, these findings suggest that fucoxanthin may modulate the gut microbiota–bile acid axis and thereby contribute to improved metabolic homeostasis.

Fucoxanthin has also been reported to inhibit carbohydrate-digesting enzymes. Yin et al. [154] demonstrated dose-dependent inhibition of α-amylase by fucoxanthin using QCM-A analysis, suggesting its potential to reduce starch breakdown in the intestine. Recent computational studies further support the potential role of fucoxanthin derivatives in modulating intestinal carbohydrate digestion. A molecular docking and molecular dynamics study reported a novel fucoxanthin derivative from *Chnoospora minima* with higher predicted binding affinity toward α-amylase and α-glucosidase than the reference drug acarbose, suggesting potential inhibitory interactions with key digestive enzymes involved in postprandial glucose regulation [155].

In addition, fucoxanthin has been shown to modulate intestinal glucose transport. In mice fed an HFHS diet, oral administration of fucoxanthin (150 mg/kg body weight/day for 8 weeks) improved hyperglycemia and insulin resistance [156]. The hypoglycemic effects were accompanied by decreased protein expression of SGLT1 as well as apical translocation of GLUT2 in the intestine. The changes in SGLT1 expression and GLUT2 translocation were associated with modulation of several signaling pathways, including the insulin/PI3K/AKT/mechanistic target of rapamycin (mTOR) and protein kinase A/AMP-activated protein kinase (AMPK)/mTOR pathways. These findings suggest that fucoxanthin may reduce intestinal glucose absorption through regulation of glucose transporters.

Despite these promising findings, evidence regarding the intestinal mechanisms of fucoxanthin remains limited. In particular, evidence for its effects on intestinal glucose transporters and the links between microbiota-mediated changes and metabolic regulation remains limited. Moreover, direct evidence for its role in incretin hormone secretion, including GLP-1 and GIP, remains scarce. Therefore, further studies using well-established T2D models and clinical trials are warranted to clarify its role in regulating intestinal glucose absorption and gut hormone-mediated metabolic pathways.

### 3.3. Hepatic Glucose and Lipid Metabolism

The liver is a central metabolic organ that regulates whole-body glucose and lipid homeostasis by integrating hormonal, nutritional, and gut-derived signals [157,158,159]. Under fed conditions, elevated insulin suppresses hepatic gluconeogenesis while promoting glycogenesis and glycolysis. In contrast, during fasting, increased glucagon stimulates gluconeogenesis and glycogenolysis to maintain blood glucose levels. In addition to these hormonal controls, gut-derived signals, including nutrients, bile acids, and microbiota-derived metabolites, further modulate hepatic metabolism through the gut–liver axis, a bidirectional communication network linking the intestine and liver [159]. Dysregulation of this axis, driven by microbiota dysbiosis, impaired intestinal barrier function, and inflammatory signaling, can contribute to hepatic insulin resistance and diabetes-related metabolic dysfunction [159]. In parallel, the liver plays a central role in lipid metabolism by regulating de novo lipogenesis, fatty acid β-oxidation, and very-low-density lipoprotein secretion.

**Figure 4:**
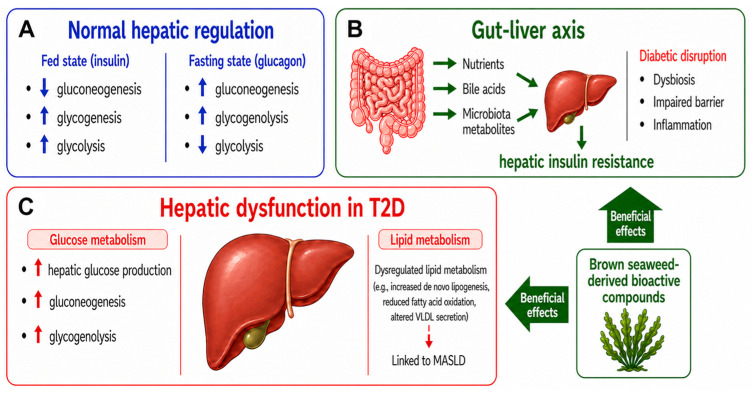
Proposed hepatic glucose and lipid metabolism-related mechanisms underlying the antidiabetic effects of brown seaweeds and their bioactive compounds. (**A**) Under normal physiological conditions, hepatic glucose metabolism is regulated differently in the fed and fasting states. (**B**) Hepatic metabolism is modulated through the gut–liver axis by gut-derived signals, including nutrients, bile acids, and microbiota-derived metabolites. Dysregulation of this axis in diabetes contributes to hepatic insulin resistance. (**C**) In T2D, dysregulation of hepatic glucose and lipid metabolism contributes to hyperglycemia and MASLD. Brown seaweed-derived bioactive compounds may help ameliorate these metabolic abnormalities.

In T2D, hepatic insulin resistance leads to excessive hepatic glucose production via increased gluconeogenesis and impaired suppression of glycogenolysis, and dysregulated lipid metabolism promotes hepatic steatosis and lipotoxicity [157,160]. These abnormalities contribute not only to fasting hyperglycemia but also to the development of MASLD which frequently coexists with T2D [161,162]. Accumulating evidence indicates that bioactive compounds derived from brown seaweeds, including fucoxanthin, fucoidan, and phlorotannins, can beneficially modulate hepatic glucose and lipid metabolism through multiple mechanisms. These mechanisms include regulation of transcription factors involved in metabolic gene expression, enhancement of insulin signaling pathways, attenuation of oxidative stress and inflammation, and improvement of mitochondrial function and energy metabolism. Moreover, these beneficial effects are partly mediated through the gut–liver axis, involving modulation of bile acid signaling and gut microbiota-derived metabolites. These hepatic metabolic abnormalities and the potential modulatory effects of brown seaweed-derived bioactive compounds are summarized in Figure 4 and further discussed in the following sections.

#### 3.3.1. Fucoidan

Multiple preclinical studies indicate that fucoidan influences hepatic glucose and lipid metabolism through several mechanisms, including suppression of hepatic gluconeogenesis and enhancement of hepatic glycolysis and glycogen synthesis, attenuation of oxidative stress and inflammation, and regulation of gut–liver axis interactions [127,128,132,163,164].

In T2D *db/db* mice, combined supplementation with low-Mw fucoidan and fucoxanthin, as well as low-Mw fucoidan alone, improved glucose tolerance and insulin resistance [163]. These effects were accompanied by increased hepatic glycogen content, glucose utilization and insulin responsiveness as well as reduced markers of hepatic damage and oxidative stress, reflecting improved glucose homeostasis and hepatoprotection in T2D mice. Notably, the metabolic improvements were more pronounced than those observed with fucoxanthin alone.

Moreover, in HFD/STZ-induced T2D rats, fucoidan derived from *Kjellmaniella crassifolia* upregulated mRNA expression of key hepatic glucose metabolism-related genes, including insulin receptor, glucokinase, and GLUT2 while reducing hepatic oxidative stress, suggesting enhanced hepatic glucose utilization and insulin responsiveness [164]. In parallel, fucoidan modulated hepatic insulin signaling, at least in part, by restoring the insulin receptor substrate (IRS)–PI3K–AKT pathway [132]. The IRS–PI3K–AKT signaling pathway plays a central role in insulin-mediated regulation of hepatic glucose metabolism [124]. Insulin binding to its receptor induces phosphorylation of IRS, which activates PI3K and subsequently AKT signaling. In the liver, activated AKT suppresses gluconeogenesis and promotes glycogen synthesis. Moreover, fucoidan decreased TLR4 and NF-κB expression in the liver, suggesting attenuation of hepatic inflammation [132]. Collectively, these findings suggest that fucoidan enhances hepatic insulin sensitivity while attenuating inflammation-associated insulin resistance. Similarly, fucoidan isolated from *Sargassum fusiforme* alleviated HFD-induced insulin resistance in mice by restoring hepatic insulin signaling, as evidenced by increased AKT phosphorylation, while concurrently activating the Nrf2/ARE antioxidant pathway and suppressing NF-κB-mediated inflammatory signaling [128].

In addition to its effects on hepatic glucose metabolism, fucoidan also markedly reduced fatty acid-induced lipid deposition in hepatocyte-like spheroid models and HepG2 cells [165]. This effect was associated with activation of the PI3K/AKT and Nrf2 signaling pathways, leading to reduced oxidative stress and suppression of NF-κB-mediated inflammatory responses. The PI3K/AKT signaling pathway is known to regulate lipid metabolism by suppressing lipogenesis and promoting fatty acid oxidation, processes that are often impaired in MASLD [166]. Consistently, in HFD- and STZ-treated mice, fucoidan derived from *Laminaria japonica* improved hyperglycemia, hyperlipidemia, and glucose tolerance while reducing hepatic lipid accumulation [127]. These metabolic improvements correlated with alterations in gut microbial taxa and associated metabolite profiles, suggesting a gut–liver axis-mediated enhancement of hepatic metabolic regulation.

Although evidence in human T2D remains limited, current preclinical studies provide a mechanistic framework suggesting that fucoidan may modulate metabolic dysfunction associated with T2D. However, direct evidence regarding its regulation of hepatic gluconeogenesis remains scarce. Further studies are therefore required to clarify whether fucoidan or fucoidan-containing brown seaweeds can directly influence these key hepatic metabolic processes.

#### 3.3.2. Alginate

Emerging evidence suggests that alginate and alginate-derived oligosaccharides may improve hepatic glucose and lipid metabolism by suppressing hepatic gluconeogenesis and lipogenesis, enhancing fatty acid β-oxidation, and modulating the gut microbiota, oxidative stress, and bile acid signaling. In a gestational diabetes mouse model, alginate oligosaccharide supplementation significantly downregulated mRNA expression of key enzymes involved in gluconeogenesis, including phosphoenolpyruvate carboxykinase (PEPCK) and glucose-6-phosphatase (G6Pase), while enhancing hepatic antioxidant defense system through activation of the Nrf2/HO-1 signaling pathway [167]. Alginate oligosaccharide also improved gut microbiota composition. However, because this evidence derives from a gestational diabetes model, its relevance to T2D should be interpreted with caution.

Beyond glucose metabolism, sodium alginate has been reported to alleviate hepatic steatosis in experimental fatty liver models [168,169], although evidence in T2D models remains limited. Zhang et al. [168] demonstrated that oral administration of sodium alginate attenuated hepatic lipid accumulation induced by a high-fat and high-cholesterol diet in rats. Its anti-steatotic effects were associated with downregulated mRNA expression of hepatic lipogenic genes, including sterol regulatory element-binding protein-1c (SREBP-1c), acetyl-CoA carboxylase (ACC), fatty acid synthase (FAS), and stearoyl-CoA desaturase-1 (SCD1), along with enhanced fatty acid β-oxidation. Moreover, these changes were accompanied by improvements in gut microbiota composition, indicating that alginate supplementation may ameliorate hepatic lipid metabolic disturbances, at least in part, through modulation of the gut microbiota. Consistently, sodium alginate supplementation attenuated hepatic lipid accumulation and improved liver histology in other experimental models of fatty liver disease, including methionine- and choline-deficient diet-induced hepatic steatosis [169].

Unsaturated alginate oligosaccharides (UAOS), another class of alginate-derived oligosaccharides, may also affect hepatic metabolism via gut–liver axis-associated bile acid signaling pathways [170]. In a mouse model of non-obese MASLD, UAOS ameliorated insulin resistance and hepatic steatosis, and these effects were associated with improved intestinal barrier function, altered gut microbial composition, changes in fecal bile acid profiles, and modulation of the FGF15/FGFR4/CYP7A1 signaling pathway.

Taken together, current evidence indicates that alginate and alginate-derived oligosaccharides may improve hepatic glucose and lipid metabolism partly through suppression of hepatic gluconeogenesis, as well as indirect mechanisms involving modulation of the gut microbiota and bile acid signaling. However, most of the available evidence comes from gestational diabetes or MASLD models rather than from T2D models. Therefore, further in vitro and in vivo studies using various experimental models of T2D are required to clarify whether alginate can directly regulate hepatic glucose and lipid metabolism. Well-designed clinical studies are also needed to determine the translational relevance of these findings in humans.

#### 3.3.3. Phlorotannins

Current evidence suggests that phlorotannins derived from brown seaweeds may exert beneficial effects on hepatic glucose and lipid metabolism in diabetic conditions. Among them, dieckol, a representative phlorotannin isolated from *E. cava*, has been reported to improve glucose homeostasis in experimental diabetic models. In an alloxan-induced hyperglycemic zebrafish model, dieckol treatment significantly reduced blood glucose levels by suppressing hepatic G6Pase and PEPCK [171]. Moreover, supplementation with a dieckol-rich extract of *E. cava* significantly improved hyperglycemia and dyslipidemia in *db/db* mice [172]. These effects were accompanied by reduced hepatic G6Pase and PEPCK activities, along with increased glucokinase activity.

Octaphlorethol A, a phlorotannin isolated from *Ishige foliacea*, also significantly improved fasting blood glucose levels and glucose tolerance in *db/db* mice, which were associated with decreased mRNA expression of hepatic PEPCK and G6Pase [173]. Similarly, extracts of *Ishige okamurae*, a brown seaweed rich in phlorotannins, improved hyperglycemia and insulin resistance in *db/db* mice by inhibiting hepatic PEPCK and G6Pase activities and by activating glucokinase activity and increasing glycogen content in the liver [174].

In addition to their effects on glucose metabolism, phlorotannins may also improve hepatic lipid metabolism. Octaphlorethol A improved hepatic steatosis by downregulating mRNA expression of hepatic FAS, an enzyme responsible for lipogenesis and lipid droplet accumulation [173]. Similarly, a dieckol-enriched extract from *Laminaria japonica* significantly attenuated hepatic steatosis in HFD-induced obese mice [175]. The extract activated AMPK and upregulated the expression of proteins involved in hepatic fatty acid β-oxidation, such as carnitine palmitoyltransferase 1 (CPT1) and peroxisome proliferator-activated receptor α (PPARα). AMPK activation has been reported to improve hepatic steatosis by increasing fatty acid oxidation in vivo [176].

However, despite the structural diversity of phlorotannins, studies investigating the direct effects of individual phlorotannin subclasses (e.g., fucols, phlorethols, fucophlorethols, fuhalols, eckols, and carmalols) on hepatic glucose and lipid metabolism remain limited.

#### 3.3.4. Fucoxanthin

In T2D *db/db* mice, administration of fucoxanthin in combination with fucoidan significantly increased hepatic glycogen content [163]. However, fucoxanthin alone induced only a modest increase in hepatic glycogen [163]. More recent evidence has provided mechanistic insights into its hepatoprotective effects [99]. In HFD and STZ-induced T2D mice, fucoxanthin alleviated liver histopathological damage, including hepatocellular vacuolation and sinusoidal disruption. These protective effects were accompanied by decreased MDA, a marker of lipid peroxidation, and increased activities of antioxidant enzymes, including SOD, CAT, and GSH-PX, in the liver, suggesting that attenuation of oxidative stress may contribute to protection against diabetes-induced hepatic injury. In addition, fucoxanthin significantly upregulated the mRNA expression of key regulators involved in insulin signaling and glucose metabolism, including AKT, AMPK, PI3K, insulin receptor, and GLUT2, indicating improved hepatic insulin sensitivity, enhanced glucose transport, and restoration of glucose homeostasis. Notably, these metabolic and antioxidant effects were further enhanced when fucoxanthin was delivered in a mono-carrier encapsulated nanoparticle formulation (FZNP). Compared with free fucoxanthin, FZNP showed superior efficacy in glycemic control, antioxidant enzyme activity, and GLUT2 expression. This enhanced activity is likely attributable to improved stability and bioavailability conferred by nanoencapsulation, which may facilitate more efficient delivery of fucoxanthin to target tissues.

Consistent with these findings, an earlier study using an insulin-resistant animal model also demonstrated that fucoxanthin and fucoxanthin-containing extracts can modulate hepatic glucose metabolism [177]. In HFD-fed mice, both fucoxanthin and *U. pinnatifida* ethanol extract (a natural source of fucoxanthin) inhibited hepatic gluconeogenic enzymes, including PEPCK and G6Pase, while activating glucokinase and increasing hepatic glycogen levels, thereby contributing to decreased fasting blood glucose and improved insulin sensitivity. These results suggest that fucoxanthin may regulate hepatic glucose homeostasis not only through insulin signaling pathways but also by modulating the balance between glucose production and utilization in the liver.

In addition to its effects on glucose metabolism, fucoxanthin has been reported to regulate hepatic lipid metabolism in both in vitro and in vivo models [71,178,179,180]. In palmitic acid-treated HepG2 cells, fucoxanthin markedly attenuated lipid deposition by downregulating lipogenic genes, including SREBP-1c and FAS, while upregulating genes involved in fatty acid β-oxidation such as PPARα and CPT1 [178]. Similar protective effects against hepatic steatosis were observed in an *ob/ob* leptin-deficient mouse model, and these effects were accompanied by activation of AMPK, a key regulator of hepatic lipid metabolism [178]. Consistently, Ye et al. [71] demonstrated that fucoxanthin alleviated oleate- and palmitate-induced lipid accumulation in hepatocytes through modulation of fatty acid oxidation and lipogenesis via AMPK signaling. In addition, attenuation of oxidative stress and inflammatory signaling pathways was associated with its hepatoprotective effects. In HFD-induced obese mice, fucoxanthin significantly reduced the activity of hepatic lipogenic enzymes, including glucose-6-phosphate dehydrogenase, malic enzyme, FAS, and phosphatidate phosphohydrolase, as well as cholesterol-regulating enzymes such as 3-hydroxy-3-methylglutaryl-CoA reductase and acyl-CoA:cholesterol acyltransferase, while increasing hepatic β-oxidation, resulting in decreased plasma and hepatic lipid levels [179]. Moreover, short-term dietary administration of fucoxanthin significantly reduced hepatic SCD1 mRNA and protein expression and altered hepatic fatty acid composition through modulation of leptin signaling in KK-A(y) mice, a T2D model characterized by severe hyperglycemia, insulin resistance, and obesity [180]. In contrast, these effects were not observed in *ob/ob* mice, suggesting a leptin-dependent mechanism.

Taken together, current evidence suggests that fucoxanthin may improve hepatic glucose and lipid metabolism and attenuate oxidative stress in T2D. However, the underlying mechanisms remain incompletely understood, particularly with respect to hepatic glucose metabolism, insulin signaling, and oxidative stress- and inflammation-related pathways. In addition, the potential involvement of the gut–liver axis requires further investigation. Well-designed clinical studies are also needed to confirm the translational relevance of these findings in humans.

### 3.4. Regulation of Glucose and Lipid Metabolism in Adipose Tissue and Skeletal Muscle

Insulin resistance in peripheral tissues such as adipose tissue and skeletal muscle is a central pathogenic feature of T2D [181]. Under normal physiological conditions, insulin promotes glucose uptake in peripheral tissues while simultaneously suppressing hepatic glucose production to maintain glucose homeostasis. Skeletal muscle accounts for approximately 70–80% of insulin-stimulated glucose uptake in the postprandial state, and impairment of this process represents a major metabolic defect in T2D [181]. Insulin-stimulated glucose uptake is primarily mediated by the insulin signaling cascade involving IRS-1, PI3K, and AKT, which ultimately regulates the translocation of glucose transporter type 4 (GLUT4) to the plasma membrane. GLUT4 is the major insulin-responsive glucose transporter in muscle and adipose tissue [182]. Upon insulin stimulation, GLUT4 translocates from intracellular vesicles to the plasma membrane, thereby facilitating glucose uptake into insulin-sensitive tissues. Dysregulation of this signaling pathway impairs GLUT4 translocation and reduces cellular glucose uptake, contributing to systemic hyperglycemia in T2D [181]. Adipose-specific deletion of the GLUT4 gene results in impaired insulin action in muscle and liver, and reduced GLUT4 expression in adipocytes disrupts whole-body glucose homeostasis [183,184].

Although adipose tissue accounts for only approximately 10% of whole-body insulin-stimulated glucose uptake, it also plays a critical role in systemic metabolic regulation by modulating lipid storage, lipolysis, and adipokine secretion [185]. Insulin stimulates glucose uptake in adipocytes by promoting GLUT4 translocation to the plasma membrane. It also exerts critical anabolic effects in adipose tissue by inhibiting lipolysis while stimulating de novo fatty acid synthesis [185,186]. In addition, insulin promotes adipocyte differentiation and lipid storage by activating transcription factors, including SREBP-1c and peroxisome proliferator-activated receptor-γ (PPARγ) [187].

AMPK, a key cellular energy sensor that regulates glucose and lipid metabolism, also plays an important role in mediating glucose uptake in peripheral tissues [188]. Activation of AMPK enhances GLUT4 translocation and increases both glucose uptake and fatty acid oxidation in skeletal muscle, thereby improving insulin sensitivity. In adipose tissue, AMPK promotes fatty acid oxidation while suppressing fatty acid synthesis and lipolysis, contributing to improved metabolic homeostasis [188].

#### 3.4.1. Fucoidan

Several studies have demonstrated that fucoidan exerts antiobesity effects through the regulation of adipose tissue metabolism [127,189]. Fucoidan protected against HFD-induced obesity in mice by decreasing the mRNA expression of genes involved in adipogenesis and lipid storage, such as PPARγ, adipocyte fatty acid-binding protein, and ACC [190]. Consistently, fucoidan derived from *U. pinnatifida* inhibited adipocyte differentiation in 3T3-L1 cells, at least in part, by downregulating adipogenic gene expression and suppressing inflammation-related cytokines [191].

Beyond its effects on adipogenesis, fucoidan also attenuated HFD-induced weight gain, adiposity, and glucose intolerance by promoting mitophagy and enhancing thermogenesis in brown adipose tissue [189]. Notably, in *db/db* mice, low-Mw fucoidan alone had minimal effects on the expression of thermogenic markers such as uncoupling protein-1 (UCP-1) in white adipose tissue (WAT), whereas co-administration with fucoxanthin significantly enhanced UCP-1 expression compared with fucoxanthin alone [163]. Similarly, low-Mw fucoidan alone did not markedly increase the mRNA expression of key insulin signaling molecules such as IRS-1 and GLUT4 in WAT, whereas its combination with fucoxanthin enhanced their expression. The combined treatment also increased adiponectin mRNA expression and decreased tumor necrosis factor (TNF)-α and interleukin (IL)-6 mRNA expression in WAT.

Unlike low-Mw fucoidan, which showed only limited anti-inflammatory effects, fucoidan derived from *Acaudina molpadioides* exerted pronounced anti-inflammatory effects when administered in a mouse model of HFHS diet-induced insulin resistance [87]. Fucoidan significantly decreased serum levels of C-reactive protein, macrophage inflammatory protein-1, IL-1β, IL-6, and TNF-α, along with increasing anti-inflammatory IL-10 levels. In parallel, it downregulated the mRNA expression of pro-inflammatory cytokines and upregulated IL-10 expression in WAT. These differences may reflect differences in fucoidan source, molecular characteristics, or experimental models.

Despite these beneficial effects, the metabolic actions of fucoidan may not be attributable solely to direct effects on peripheral tissues, but may also involve indirect mechanisms. For instance, low-Mw fucoidan did not significantly affect glucose uptake in myotubes and adipocytes [192]. Similarly, in HFD-fed pseudo-germ-free mice, fucoidan derived from *Sargassum fusiforme* improved insulin resistance without significantly increasing AKT phosphorylation in muscle or adipose tissue [129]. However, Jeong et al. [193] provided evidence that fucoidan can directly improve peripheral insulin sensitivity. Specifically, low-Mw fucoidan significantly ameliorated insulin resistance in both in vitro and in vivo models. In L6 myotubes and *db/db* mice, it alleviated ER stress-induced insulin resistance by activating AMPK signaling and restoring AKT phosphorylation, thereby increasing glucose uptake and fatty acid oxidation and improving metabolic homeostasis.

Collectively, these findings indicate that fucoidan may exert antiobesity and insulin-sensitizing effects through multiple mechanisms in adipose tissue and muscle, although the extent of its direct actions on peripheral tissues may vary depending on molecular characteristics and experimental conditions.

#### 3.4.2. Alginate

Compared with fucoidan, direct evidence supporting a role for alginate in regulating glucose and lipid metabolism in adipose tissue and skeletal muscle remains relatively limited, particularly for native alginate. Most reported metabolic effects of alginate have been attributed to gastrointestinal and microbiota-related mechanisms, including modulation of gastric function, digestive processes, and gut microbial composition. However, recent studies, particularly those using alginate oligosaccharides, suggest that direct effects on peripheral tissues may also contribute to its metabolic effects [194,195].

#### 3.4.3. Phlorotannins

In addition to their effects on hepatic metabolism, phlorotannins have been reported to modulate glucose metabolism in skeletal muscle. In an alloxan-induced hyperglycemic zebrafish model, dieckol isolated from *E. cava* increased AKT phosphorylation in muscle tissue, thereby improving insulin sensitivity and promoting glucose uptake [171]. Consistently, the antidiabetic effects of *E. cava* dieckol were associated with increased phosphorylation of AMPK and AKT in skeletal muscle of T2D *db/db* mice [196]. In line with these findings, *E. cava* methanol extract activated both AMPK and PI3K/AKT signaling pathways in C2C12 myoblasts [197]. Further supporting these observations, dieckol derived from *E. cava* enhanced glucose uptake in L6 muscle cells in a dose-dependent manner, accompanied by activation of the PI3K/AKT and AMPK signaling pathways, as evidenced by the attenuation of this effect following treatment with the PI3K inhibitor wortmannin and the AMPK inhibitor compound C [198]. Notably, this effect occurred independently of insulin and was associated with increased GLUT4 translocation to the plasma membrane. These findings suggest that the PI3K/AKT and AMPK signaling pathways contribute to the regulation of glucose uptake in skeletal muscle.

AMPK plays a central role in cellular energy homeostasis by coordinating anabolic and catabolic pathways in a tissue-specific manner. In skeletal muscle, AMPK activation promotes glucose uptake, thereby enhancing metabolic flexibility and insulin sensitivity [199,200]. By contrast, in adipose tissue, AMPK activation generally suppresses lipogenesis and can inhibit adipocyte differentiation by downregulating key lipogenic enzymes and adipogenic transcription factors [201,202]. In 3T3-L1 preadipocytes, dieckol, a phlorotannin isolated from *E. cava*, inhibited adipocyte differentiation through activation of AMPK [203]. Dieckol treatment also suppressed the expression of key adipogenic transcription factors, including PPARγ, CCAAT/enhancer binding protein (C/EBP)α, and SREBP-1c, leading to reduced lipid accumulation.

Similarly, octaphlorethol A, a phlorotannin isolated from *Ishige foliacea*, has been reported to exert antidiabetic effects by improving peripheral glucose metabolism [173,204]. In differentiated L6 rat myoblast cells, octaphlorethol A stimulated GLUT4-mediated glucose uptake in a dose-dependent manner through coordinated activation of the PI3K/AKT and AMPK signaling pathways [204]. Consistent with these in vitro findings, octaphlorethol A enhanced AMPK phosphorylation and increased GLUT4 expression in skeletal muscle of *db/db* mice, thereby promoting glucose uptake and improving insulin sensitivity [173].

More recently, Xiao et al. [96] reported that trifuhalol A, a phlorotannin derived from *Agarum cribrosum*, enhanced glucose uptake in C2C12 myotubes via activation of the PI3K/AKT and AMPK signaling pathways, which in turn promoted GLUT4 translocation to the plasma membrane. This may contribute to enhanced glucose utilization and improved insulin sensitivity. Similarly, diphlorethohydroxycarmalol (DPHC), a phlorotannin from *Ishige okamurae*, increased cytosolic Ca^2+^ levels in skeletal muscle of alloxan-treated zebrafish larvae, which was associated with activation of AMPK activation and enhanced GLUT4-mediated glucose transport [205]. Elevated cytosolic Ca^2+^ can trigger muscle contraction, generating signals that promote GLUT4 translocation to the plasma membrane [206,207]. Together, these findings suggest that DPHC may enhance glucose transport in skeletal muscle and contribute to improved blood glucose regulation.

In addition to the activation of PI3K/AKT and AMPK signaling pathways, phlorotannins are reported to regulate protein tyrosine phosphatase 1B (PTP1B), a negative regulator of insulin signaling [208]. Several phlorotannins isolated from *Ecklonia stolonifera* and *Eisenia bicyclis* (e.g., eckol, phlorofucofuroeckol A, dieckol, and 7-phloroeckol) exhibited potent PTP1B inhibitory activity with low micromolar IC_50_ values, suggesting their potential to enhance insulin sensitivity by alleviating negative regulation of insulin signaling pathways [146].

Collectively, current evidence indicates that phlorotannins may modulate multiple signaling pathways involved in insulin sensitivity, glucose uptake and lipid metabolism in peripheral tissues, particularly through coordinated regulation of PI3K/AKT, AMPK, Ca^2+^-dependent signaling, and PTP1B inhibition. However, despite these promising findings, most studies remain limited to in vitro systems or animal models, and the relative contribution of each pathway to overall metabolic regulation has not been fully elucidated. In particular, further studies are needed to clarify tissue-specific mechanisms, dose–response relationships, and structure–activity relationships among different phlorotannin subclasses. Moreover, well-designed preclinical studies using clinically relevant T2D models, together with human trials, are required to validate these effects and establish their translational potential for metabolic disease management.

#### 3.4.4. Fucoxanthin

Fucoxanthin has been reported to improve insulin sensitivity in peripheral tissues. In *db/db* mice, fucoxanthin significantly upregulated the mRNA expression of IRS-1 and GLUT4 in WAT, suggesting enhanced insulin signaling and glucose uptake [163]. In addition, fucoxanthin increased the mRNA expression of PPARγ and UCP-1 in adipose tissue. Activation of PPARγ, a key regulator of adipocyte differentiation and lipid storage, has been reported to improve insulin sensitivity and induce the expression of mitochondrial uncoupling proteins such as UCP-1 [209,210]. Although UCP-1 is a hallmark of brown adipocytes and plays an important role in cold- and diet-induced thermogenesis, its expression in WAT is also implicated in energy balance and obesity development in both animals and humans [211,212]. Therefore, fucoxanthin-induced UCP-1 expression may contribute to enhanced energy expenditure and antiobesity effects, which are closely linked to improved insulin sensitivity, as supported by previous studies [213,214]. Consistent with the role of fucoxanthin in enhancing energy metabolism in adipose tissue, Kang et al. [60] reported that fucoxanthin increased AMPK and ACC phosphorylation, as well as CPT-1a mRNA expression, in mature 3T3-L1 adipocytes, indicating enhanced fatty acid oxidation. Collectively, these findings suggest that the antiobesity effects of fucoxanthin may be associated with UCP-1 induction and enhanced fatty acid oxidation, which may contribute to improved insulin sensitivity and increased glucose uptake in adipose tissue.

However, the effects of fucoxanthin on glucose and lipid metabolism in adipocytes appear to be differentiation-stage-dependent. In 3T3-L1 preadipocytes, fucoxanthin promoted adipocyte differentiation during the early stage, accompanied by increased expression of adipogenic markers, including PPARγ and C/EBPα, whereas it suppressed lipid accumulation and downregulated adipogenic regulators at later stages [60]. In mature adipocytes, fucoxanthin inhibited insulin-stimulated glucose uptake by decreasing IRS-1 phosphorylation, indicating that its metabolic effects may be context-dependent and vary according to the stage of adipocyte differentiation [60].

Regulation of GLUT4 expression by fucoxanthin in skeletal muscle is also associated with its antidiabetic effects [16,215]. In HFD-fed mice, dietary fucoxanthin (in the form of fucoxanthin-rich wakame lipids) restored the reduced GLUT4 mRNA expression in skeletal muscle to near normal levels, accompanied by normalization of hyperglycemia and hyperinsulinemia [215]. Consistent with these findings, Nishikawa et al. [16] demonstrated that fucoxanthin promoted GLUT4 translocation and increased GLUT4 expression in skeletal muscle of KK-A(y) mice. This effect was accompanied by increased expression of peroxisomal proliferator-activated receptor-γ coactivator-1α, a key transcriptional coactivator that regulates energy metabolism and linked to the regulation of GLUT4 expression [216], as well as increased insulin receptor mRNA expression and enhanced AKT phosphorylation, collectively indicating activation of insulin signaling pathways and thereby improved glucose uptake and glycemic control.

Adipose tissue is widely recognized as an active endocrine organ that secretes numerous biologically active mediators (adipocytokines), including leptin, adiponectin, resistin, TNF-α, IL-6, monocyte chemoattractant protein-1 (MCP-1), and plasminogen activator inhibitor-1 (PAI-1) [217]. Some adipocytokines modulate insulin sensitivity as well as glucose and lipid metabolism in adipose tissue, muscle, and liver [217]. Dysregulation of adipocytokines, including decreased adiponectin and increased leptin, resistin, and pro-inflammatory cytokines, contributes to insulin resistance and T2D [218]. Hosokawa et al. [219] demonstrated that fucoxanthin attenuated obesity and hyperglycemia and downregulated the mRNA expression of MCP-1, TNF-α, IL-6, and PAI-1 in the WAT of T2D KK-Ay mice, but not in lean C57BL/6J mice. In addition, fucoxanthinol, a metabolite of fucoxanthin detected in adipose tissue, attenuated TNF-α-induced MCP-1 and IL-6 expression in differentiating adipocytes [216]. Pro-inflammatory markers, such as MCP-1, TNF-α, and IL-6, are elevated in T2D and contribute to adipose inflammation and insulin resistance [220]. In particular, treatment of differentiated adipocytes with MCP-1 decreased insulin-stimulated glucose uptake and reduced the expression of adipogenic genes such as GLUT4 and PPARγ [221]. PAI-1 mRNA levels were also elevated in obese adipose tissue and were associated with obesity, insulin resistance, thrombosis, and fibrosis [222]. Collectively, these findings suggest that fucoxanthin-mediated regulation of adipocytokines may contribute to improved insulin resistance. This is supported by a previous study showing that dietary fucoxanthin, administered as fucoxanthin-rich wakame lipids, downregulated MCP-1 mRNA expression in WAT of HFD-induced obese mice, along with increased GLUT4 expression in skeletal muscle [215].

Hosokawa et al. [219] further demonstrated that fucoxanthinol decreased TNF-α, inducible nitric oxide synthase (iNOS), and cyclooxygenase-2 (COX-2) mRNA expression in palmitic acid-stimulated RAW264.7 macrophage-like cells. iNOS produces nitric oxide, a reactive molecule involved in inflammatory pathogenesis, and COX-2 catalyzes the conversion of arachidonic acid into pro-inflammatory prostaglandins. Both enzymes are induced by pro-inflammatory stimuli, including cytokines and pathogens. Pharmacological inhibition of COX-2 enhanced insulin sensitivity by reversing defects in glycogen synthase activity and GLUT4 translocation in skeletal muscle [223]. Therefore, the downregulation of iNOS and COX-2 by fucoxanthinol, a metabolite of fucoxanthin, may contribute to the amelioration of insulin resistance through suppression of inflammatory signaling.

Taken together, current evidence indicates that fucoxanthin exerts beneficial effects on glucose and lipid metabolism in peripheral tissues through multiple mechanisms, including enhancement of insulin signaling, activation of AMPK-mediated energy metabolism, induction of thermogenic pathways, and attenuation of inflammation. However, the metabolic effects of fucoxanthin appear to be context-dependent, varying according to cell type, differentiation stage, and experimental conditions. In particular, conflicting findings regarding its effects on adipocyte glucose uptake highlight the need for further mechanistic clarification. Moreover, because most studies have been conducted in vitro or in animal models, well-designed studies using clinically relevant models, together with human trials, are required to validate these findings. Future research should also focus on elucidating tissue-specific mechanisms and the interplay between metabolic and inflammatory pathways to better understand the therapeutic potential of fucoxanthin in T2D.

## 4. Bioavailability

Bioavailability is an important determinant of the translational potential of brown seaweed-derived bioactive compounds after oral administration. Available evidence indicates that the absorption, metabolism, and systemic or tissue distribution of these compounds vary substantially depending on their chemical structures and physicochemical properties.

Fucoidan, a high-Mw sulfated polysaccharide, has generally been considered to have limited intestinal absorption. However, pharmacokinetic studies suggest that orally administered fucoidan can be detected in the circulation, although concentrations vary depending on Mw, algal source, dose, duration of administration, and analytical method [224,225,226]. After a single intragastric administration of *F. vesiculosus* fucoidan (Mw 735 kDa) at a dose of 100 mg/kg in rats, fucoidan was detected in plasma and preferentially accumulated in the kidneys, spleen, and liver [224]. The maximum concentrations were 0.125 mg/L in plasma at 4 h, 1.23 μg/g in the kidney at 5 h, 0.78 μg/g in the spleen at 3 h, and 0.53 μg/g in the liver at 2 h [224]. In humans, fucoidan was not detected in serum before administration, whereas serum fucoidan levels markedly increased at 6 and 9 h after oral administration of 1 g of *Cladosiphon okamuranus* fucoidan [227]. Another human study using a competitive ELISA reported detectable plasma fucoidan following 12 days of repeated oral intake of 3 g/day of *Undaria pinnatifida* extracts containing either 10% or 75% fucoidan (Mw 713 kDa) [226]. The median plasma concentrations were 4.002 and 12.989 mg/L in the 10% and 75% fucoidan groups, respectively. Nevertheless, the overall intestinal absorption of fucoidan appears to be low, and its systemic availability should be interpreted cautiously because of differences in molecular size, source, dose regimen, and detection method.

Compared with fucoidan, another major brown seaweed-derived polysaccharide alginate is unlikely to exert its major metabolic effects through systemic absorption. As a dietary fiber, alginate and its oligosaccharides are largely retained in the gastrointestinal tract, although a small fraction of alginate oligosaccharides may be absorbed and excreted in urine. For example, sodium alginate oligosaccharides were substantially recovered in feces, while a smaller proportion was detected in urine after oral administration [228]. Another LC–MS/MS study reported that alginate oligosaccharides, at least from dimer to tetramer, were rapidly detected in mouse plasma and urine after a single oral dose [227]. In plasma and urine, the maximum concentrations were 24.5 mg/L at 5 min and 425.5 mg/L at 30 min after oral administration, respectively [227]. Alginate oligosaccharides were no longer detected in plasma at 2 h, but remained detectable in urine up to 6 h at low levels [227].

Phlorotannins also show limited and structure-dependent bioavailability. In a human study using *Ascophyllum nodosum* phlorotannin-rich extract, unconjugated and conjugated metabolites were detected in urine and plasma, mainly at late time points (6–24 h), suggesting that high-Mw phlorotannins are poorly absorbed in the upper gastrointestinal tract and may reach the large intestine, where they are metabolized into lower-Mw derivatives by the colonic microbiota [229]. More recent pharmacokinetic data in rats showed limited detectability and low systemic availability of major *Ecklonia cava* phlorotannins, including dieckol, 8,8′-bieckol, and phlorofucofuroeckol-A, after oral administration [230].

Fucoxanthin differs from the polysaccharides and phlorotannins in that it is absorbed mainly after metabolic conversion. In humans, oral administration of kombu extract containing 31 mg fucoxanthin resulted in detectable plasma fucoxanthinol, but not fucoxanthin itself, with a maximum concentration of 44.2 nmol/L at 4 h, and a terminal half-life of 7.0 h [231]. The hepatic metabolite of fucoxanthinol, amarouciaxanthin A, was not detected in human plasma [231].

These findings indicate that the therapeutic potential of brown seaweed-derived compounds should be interpreted in light of their limited, compound-specific, and often metabolite-mediated bioavailability.

## 5. Toxicological Considerations

Toxicological information is also important for evaluating the therapeutic applicability of brown seaweed-derived bioactive compounds. At present, specific toxic threshold concentrations in humans have not been clearly established for all four major compounds. Therefore, safety evaluation relies mainly on available NOAELs, repeated-dose toxicity studies, regulatory intake levels, and human tolerability data.

For fucoidan, low-Mw fucoidan from *Laminaria japonica* showed no mutagenicity in vitro at 5000 μg/mL and produced no toxicological indications in rats at 2000 mg/kg body weight/day [232]. Fucoidan from *Cladosiphon okamuranus* Tokida has also been evaluated in a randomized, double-blind, parallel-group, placebo-controlled pilot study in healthy adults, in which 3.0 g/day fucoidan was administered as a beverage for 12 weeks and no test food-related adverse events were observed [233]. For alginate, the EFSA re-evaluation of alginic acid and its salts concluded that there was no need for a numerical acceptable daily intake and no safety concern at the refined exposure estimates for their reported food additive uses [234]. For *Ecklonia cava* phlorotannins, EFSA concluded that the novel food is safe for use in food supplements at maximum daily intake levels of 263 mg/day for adults, 230 mg/day for adolescents above 14 years, and 163 mg/day for adolescents aged 12–14 years [235]. In addition, preclinical toxicity studies reported no mortality or major treatment-related toxicological changes after single oral administration of *E. cava* extract containing 65% phlorotannins at 2000 mg/kg body weight, or after repeated oral administration of *E. cava* extract containing 20% phlorotannins at doses up to 2000 mg/kg/day for 4 weeks in rats [236]. For fucoxanthin, single and repeated oral-dose toxicity studies in mice reported no mortality or abnormalities in gross appearance at the tested doses (single doses of 1000 and 2000 mg/kg body weight and repeated doses of 500 and 1000 mg/kg body weight/day for 30 days) [237]. In addition, a fucoxanthin-containing nutraceutical preparation (Xanthigen^®^, Nektium Pharma S.L., Agüimes, Spain; a combination of *Undaria pinnatifida* extract and pomegranate seed oil) showed no mortality or significant treatment-related adverse effects at oral doses up to 2000 mg/kg and had a 90-day NOAEL of at least 1000 mg/kg/day in rats [237,238].

Overall, these data suggest a favorable safety profile at experimentally or regulatorily evaluated doses, but they do not define universal toxic threshold concentrations. Differences in compound purity, Mw, algal source, formulation, exposure duration, and iodine/heavy-metal contamination in seaweed-derived preparations should be considered when extrapolating these findings to humans.

## 6. Clinical Evidence

Despite evidence from in vitro and animal studies, the clinical relevance of brown seaweeds and their bioactive compounds in T2D requires careful evaluation. Although a few human studies have investigated the effects of brown seaweeds and their bioactive compounds on metabolic health, direct clinical evidence in individuals with T2D remains limited and somewhat inconsistent.

For instance, the antidiabetic potential of *E. cava*-derived phlorotannins has been explored in humans. In a randomized, double-blind, placebo-controlled clinical trial involving prediabetic individuals, supplementation with a dieckol-rich extract of *E. cava* significantly reduced postprandial blood glucose levels compared with the placebo group [37].

In contrast, clinical evidence for alginate shows more limited metabolic effects. In a randomized controlled trial involving obese subjects, alginate supplementation (15 g/day, administered as a preload before meals) was evaluated over a 12-week energy-restricted diet (300 kcal/day deficit) [239]. Although no significant differences in body weight reduction were observed between groups when all participants were included in the analysis, alginate supplementation modestly enhanced weight loss and reduced body fat compared with the placebo group among participants who completed the intervention. However, alginate supplementation did not significantly affect glycemic parameters, including plasma glucose, insulin, or HOMA-IR, nor did it improve lipid profiles or inflammatory markers.

A concise summary of key clinical studies evaluating brown seaweed-based interventions relevant to T2D and metabolic health is provided in Table 1.

As summarized in Table 1, the available clinical evidence is difficult to compare directly because of substantial heterogeneity in study population, intervention composition, dosage, treatment duration, and outcome measures. Some studies enrolled individuals with established T2D, whereas others included individuals with prediabetes, obesity, or metabolic syndrome. Moreover, several interventions used whole seaweed extracts or combined nutraceutical formulations, making it difficult to attribute the observed effects to a specific compound. Differences in bioavailability may further contribute to inconsistent outcomes, given that alginate primarily exerts gastrointestinal effects, fucoidan and phlorotannins show limited and structure-dependent systemic availability, and fucoxanthin is mainly detected as metabolites after oral intake. Therefore, future clinical studies should use standardized compound-specific preparations, clearly defined T2D populations, appropriate dosing regimens, and clinically relevant endpoints such as HbA1c, fasting and postprandial glucose, insulin sensitivity, and long-term safety.

Overall, the available clinical evidence remains preliminary and insufficient to establish the therapeutic efficacy of brown seaweed-derived bioactive compounds in individuals with T2D. Therefore, the promising findings from preclinical studies should be interpreted cautiously until they are confirmed well-designed randomized controlled trials in individuals with T2D.

## 7. Future Perspectives

Given the limitations of current clinical and preclinical studies, several critical challenges remain for the clinical translation of these bioactive compounds. First, most current evidence is derived from in vitro and animal studies, and well-designed human clinical trials are still limited. Second, the bioavailability, metabolic stability, and tissue-specific distribution of these compounds are not yet fully understood, particularly for structurally complex molecules such as phlorotannins and fucoidan. Third, considerable variability in chemical composition, depending on seaweed species, extraction methods, and molecular characteristics (e.g., Mw, degree of sulfation, and degree of polymerization), poses challenges for standardization and reproducibility across studies.

Furthermore, although multiple signaling pathways, including PI3K/AKT, AMPK, and inflammatory cascades, have been implicated, the precise molecular targets and their interactions within organ-specific and inter-organ metabolic networks remain to be fully elucidated. In particular, emerging evidence suggests a potential role of the gut–liver axis, bile acid signaling, and gut microbiota-derived metabolites in mediating the metabolic effects of these compounds. However, these mechanisms are still not sufficiently characterized in well-established T2D models and human studies.

Therefore, future research should focus on (1) elucidating structure–activity relationships of individual bioactive compounds, (2) improving their bioavailability and delivery systems (e.g., nanoformulations), (3) conducting well-controlled clinical trials to validate efficacy and safety in humans, and (4) integrating multi-omics approaches to better understand their systemic and tissue-specific metabolic effects. Addressing these gaps will be essential to advance the development of brown seaweed-derived bioactive compounds from experimental observations to clinically relevant nutritional strategies for T2D management.

## 8. Conclusions

This review provides an overview of the antidiabetic potential of brown seaweeds, with a particular focus on the mechanisms of action of their major bioactive compounds. The ability of fucoidan, alginate, phlorotannins, and fucoxanthin to regulate multiple pathophysiological processes, including pancreatic β-cell function, insulin signaling, glucose and lipid metabolism, oxidative stress, inflammation, and gut-mediated metabolic pathways, suggests their potential to improve T2D-associated metabolic dysregulation. However, these findings are derived predominantly from in vitro and animal studies, and current clinical evidence remains insufficient to establish therapeutic efficacy in people with T2D. Therefore, further well-designed, compound-specific clinical trials in individuals with T2D are required to confirm clinically meaningful effects, define effective doses, and evaluate long-term safety.

## Figures and Tables

**Table 1:** Summary of key clinical studies on brown seaweed-based interventions relevant to T2D and metabolic health.

Intervention	Study Design	Population	Dosage/Duration	Main Outcomes	Main Limitations
Dieckol-rich *Ecklonia cava* extract [37]	Randomized, double-blind, placebo-controlled, parallel-group trial	80 prediabetic adults	1500 mg/day for 12 weeks	Reduced postprandial glucose compared with placebo	Prediabetic, not established T2D; limited duration; extract-based intervention rather than isolated dieckol
High-Mw fucoidan beverage [133]	Randomized, double-blind, placebo-controlled crossover trial	30 patients with T2D managed by diet therapy	60 mL/day beverage containing 1620 mg fucoidan or placebo during two 12-week intervention periods, separated by a 4-week washout interval	No significant improvement in overall glycemic control; significant reductions in HbA1c and baseline GLP-1 were observed only in a subgroup with normal HOMA-IR (<2.5).	Small sample size; crossover design; subgroup effect; diet-treated T2D population; limited generalizability
Sodium alginate [46]	Randomized acute meal-based crossover study	7 patients with well-controlled T2D	Meal with 5.0 g sodium alginate	Delayed gastric emptying and lowered postprandial blood glucose, insulin, and C-peptide responses	Very small sample; acute postprandial study; no assessment of long-term glycemic control
Alginate supplementation [239]	Randomized, double-blind, placebo-controlled, parallel-group trial	Obese subjects undergoing an energy-restricted diet	Alginate-based preload beverage providing 15 g fiber/day, administered 3 times/day before main meals for 12 weeks	Greater weight loss and body fat reduction in participants who completed the intervention; no significant effects on plasma glucose, insulin, HOMA-IR, lipid profiles, or inflammatory markers	Obese, not T2D; effects mainly in completer analysis; intervention conducted during energy restriction
*Ascophyllum nodosum*, *Fucus vesiculosus* extract plus chromium picolinate [45]	Multicenter, randomized, double-blind, placebo-controlled trial	175 overweight patients with T2D on stable antidiabetic therapy	Nutraceutical formulation containing *A. nodosum* and *F. vesiculosus* extract and chromium picolinate for 6 months	Reduced HbA1c, fasting plasma glucose, and postprandial glucose compared with placebo	Combined formulation; effects cannot be attributed solely to brown seaweed extract; background antidiabetic therapy; contribution of chromium cannot be separated
Fucoxanthin [103]	Randomized, double-blind, placebo-controlled trial	28 patients with metabolic syndrome	12 mg/day for 12 weeks	No significant change in fasting glucose; decreases in body weight, waist circumference, blood pressure, and triglycerides; increased first-phase and total insulin secretion	Metabolic syndrome, not T2D; small sample size; no HbA1c or long-term glycemic control assessment

## Data Availability

No new data were created or analyzed in this study. Data sharing is not applicable to this article.

## Abbreviations

The following abbreviations are used in this manuscript:

ACC	Acetyl-CoA carboxylase
AMPK	Adenosine monophosphate-activated protein kinase
CaMK	Calmodulin-dependent protein kinase
CVD	Cardiovascular disease
CPT1	Carnitine palmitoyltransferase 1
C/EBPα	CCAAT/enhancer binding protein
cAMP	Cyclic AMP
COX-2	Cyclooxygenase-2
DPHC	Diphlorethohydroxycarmalol
ER	Endoplasmic reticulum
GLUT2	Glucose transporter 2
GLUT4	Glucose transporter 4
FAS	Fatty acid synthase
FUDPs	Fucoidan degradation products
FZNP	A mono-carrier encapsulated nanoparticle formulation
GLP-1	Glucagon-like peptide-1
GLP-1R	Glucagon-like peptide-1 receptor
G6Pase	Glucose-6-phosphatase
GIP	Glucose-dependent insulinotropic polypeptide
HFD	High-fat diet
HFHS	High-fat, high-sucrose
iNOS	Inducible nitric oxide synthase
IRS	Insulin receptor substrate
IL-6	Interleukin-6
mTOR	Mechanistic target of rapamycin
MASLD	Metabolic dysfunction-associated steatotic liver disease
Mw	Molecular weight
MCP-1	Monocyte chemoattractant protein-1
NF-κB	Nuclear factor kappa B
PDX-1	Duodenal homeobox-1
PPARα	Peroxisome proliferator-activated receptor α
PPARγ	Peroxisome proliferator-activated receptor-γ
PI3K	Phosphatidylinositol 3-kinase
PEPCK	Phosphoenolpyruvate carboxykinase
PAI-1	Plasminogen activator inhibitor-1
PKB	Protein kinase B
PKC	Protein kinase C
PTP1B	Protein tyrosine phosphatase 1B
SCFA	Short-chain fatty acid
Sirt1	Sirtuin-1
SGLT1	Sodium-dependent glucose transporter 1
SCD1	Stearoyl-CoA desaturase-1
SREBP-1c	Sterol regulatory element-binding protein-1c
STZ	Streptozotocin
TLR4	Toll-like receptor 4
TNF-α	Tumor necrosis factor-α
T2D	Type 2 diabetes
UCP-1	Uncoupling protein-1
UAOS	Unsaturated alginate oligosaccharides
WAT	White adipose tissue
